# Genome-Wide Dissection of MATE Gene Family in Cultivated Peanuts and Unveiling Their Expression Profiles Under Aluminum Stress

**DOI:** 10.3390/ijms26062707

**Published:** 2025-03-17

**Authors:** Saba Hameed, Xia Li, Yunyi Zhou, Jie Zhan, Aiqin Wang, Zhuqiang Han, Dong Xiao, Longfei He

**Affiliations:** 1 National Demonstration Center for Experimental Plant Science Education, College of Agriculture, Guangxi University, Nanning 530004, China; 1817401021@st.gxu.edu.cn (S.H.); 2017401005@st.gxu.edu.cn (X.L.); zhouyunyi210@163.com (Y.Z.); may2399@gxu.edu.cn (J.Z.); waiqing1966@126.com (A.W.); xiaodong@gxu.edu.cn (D.X.); 2 Guangxi Key Laboratory for Agro-Environment and Agro-Product Safety, Nanning 530004, China; 3 Guangxi Colleges and Universities Key Laboratory of Crop Cultivation and Tillage, Nanning 530004, China; 4 Cash Crops Research Institute, Guangxi Academy of Agricultural Sciences, Nanning 530004, China; hanzhuqiang@163.com

**Keywords:** peanut, genome-wide dissection, MATE gene family, protein characteristics, phylogenetic evolution, expression profiles, Al stress

## Abstract

Peanut faces yield constraints due to aluminum (Al) toxicity in acidic soils. The multidrug and toxic compound extrusion (MATE) family is known for extruding organic compounds and transporting plant hormones and secondary metabolites. However, the MATE transporter family has not yet been reported in peanuts under the Al stress condition. In this genome-wide study, we identified 111 genes encoding MATE proteins from the cultivated peanut genome via structural analysis, designated as *AhMATE1*–*AhMATE111*. Encoded proteins ranged from 258 to 582 aa residues. Based on their phylogenetic relationship and gene structure, they were classified into six distinct groups. Genes were distributed unevenly on twenty peanut chromosomes. Chr-05 exhibited the higher density of 12%, while chr-02 and chr-11 have the lowest 1% of these loci. Peanut MATE genes underwent a periodic strong to moderate purifying selection pressure during evolution, exhibiting both tandem and segmental duplication events. Segmental duplication accounted for 82% of the events, whereas tandem duplication represented 18%, with both events predominantly driving their moderate expansion. Further investigation of seven *AhMATE* genes expression profiles in peanut root tips resulted in distinct transcriptional responses at 4, 8, 12, and 24 h post-Al treatment. Notably, *AhMATE* genes exhibited greater transcriptional changes in the Al-tolerant cultivar 99-1507 compared to the Al-sensitive cultivar ZH2 (Zhonghua No.2). Our findings provide the first comprehensive genome-wide analysis of the MATE family in cultivated peanuts, highlighting their potential roles in response to Al stress.

## 1. Introduction

Peanut (*Arachis hypogaea* L.) is a major economic crop worldwide and a rich source of edible oil and nutrients [1]. China is the leading country as far as peanut production is concerned, accounting for 20% of the planting area and 40% of the yield globally [2]. Peanut cultivation land in southern China mostly consists of red and yellow soil with acidic properties. The area of acid soils with pH values lower than 6.5 is about 3.11 million km^2^, accounting for 32.4% of the total national land area in China [3]. Peanut plants are susceptible to soil acidification, which reduces their yield. Aluminum (Al) in acidic soils is a cause of decreased agricultural production worldwide [4], because Al is widely recognized as an inhibitory element for plant growth, especially in acidic soils, where the pH levels are around 5 or 5.5. Under these conditions, the most phytotoxic form of aluminum Al^3+^, becomes increasingly soluble and prevalent in the soil [5]. The increased solubility of Al^3+^ in acidic soils (pH < 5.5) initially inhibits root elongation by destroying the cell structure of the root apex, thus impairing water and nutrient uptake by the roots. This disruption of root growth and functions significantly reduces crop yields [6]. In plants, Al exclusion and internal tolerance are the two main physiological mechanisms for tolerating Al toxicity. Some membrane transporters, which either decrease Al concentrations in the cytosol or facilitate the transport of organic acids such as malate, citrate, and oxalate, are the main contributors in the exclusion mechanism [7].

The multidrug and toxic compound extrusion (MATE) family is a multidrug efflux transporter family that was categorized by Brown et al. in 1999 [8]. MATE secondary transporter proteins are a family of cation antiporters that were first identified in *Vibrio parahaemolyticus*, named as NorM, and its homolog in *Escherichia coli*, named YdhE, as multidrug efflux proteins [9]. Initially, they were categorized as a major facilitator superfamily (MFS) because, like the true MFS, NorM has 12 transmembrane regions (TMs) and may function a Na^+^/drug antiporter [10]. However, later on, these two transporters were designated as MATE due to their lack of sequence homology with other known transporters [8,9].

MATE efflux transporter families are secondary transporters that utilize sodium ion or proton gradients as their driving force. Each MATE protein functions as an electro-neutral Na^+^ or H^+^/organic cation antiporter. H^+^-driven MATE proteins are ubiquitously conserved in all three domains of life, whereas Na^+^-driven MATE proteins have been identified only in halophilic bacteria [11,12,13].

MATE transporters are widely distributed across bacteria, fungi, mammals, and plants. Omote et al. identified 861 MATE transporters from *Archaea*, *Eubacteria*, and *Eukarya*, and classified them into three major subfamilies comprising 14 subgroups [14]. Compared with the fairly small numbers of MATE members in bacteria and *Homo sapiens*, which has only two MATE genes excluding isoforms, plants exhibit a higher diversity of MATE genes [12,15]. For instance, 56 MATE genes have been identified in *Arabidopsis thaliana* [16], and 70 genes have been identified in *Medicago truncatula* [17]. MATE gene families have also been identified in other plant species, including soybean [18], maize [19], rice [16,20], tomato [21], populous [22], cotton [23], potato [24], pigeon pea [25], chickpea [26], mung bean [27], and lentil [28].

In plants, the MATE family plays crucial roles in growth and development. Among the first functionally characterized MATE efflux transporter in plants was *AtALF5* from *Arabidopsis thaliana* [29], followed by the first multi-specific MATE transporter, *AtDTX1*. Functional analysis of the protein encoded by *AtDTX1* in the *kam3* mutant demonstrated that *AtDTX1* serves as an efflux carrier for plant-derived alkaloids, antibiotics, and other toxic compounds [30]. MATE transporters involved in the detoxification of aluminum were first identified in sorghum (*SbMATE*) and barley (*HvAACT1*) via map-based cloning [31,32]. The *HvAACT1* gene encoded protein facilitates citrate release for iron translocation and improves aluminum tolerance in barley and wheat [33,34].

Compared to other plant species, research on MATE transporters in peanuts is limited. However, the availability of whole-genome sequencing data for cultivated peanut provides an opportunity to identify and characterize MATE transporters, as well as to explore their expression patterns and potential functions. This study focuses on surveying the entire peanut genome to identify and characterize potential MATE family genes, including their chromosomal distribution, gene duplication events, phylogenetic relationships, and structural features. Additionally, we aim to analyze the expression levels of several MATE genes in peanut root tips post-aluminum treatment using qRT-PCR, comparing contrasting genotypes (Al-tolerant and Al-sensitive). This research intends to facilitate future cloning and functional studies of MATE transporters in peanuts, and to enhance the understanding of peanut MATE family evolutionary history.

## 2. Results

### 2.1. Identification and Characterization of AhMATEs

A total of 111 candidate MATE gene sequences were retrieved by performing an hmmsearch against the whole-genome sequence of cultivated peanut (*Arachis hypogaea*). Based on their chromosomal locations, putative MATEs were designated as *AhMATE1*–*AhMATE111* (Supplementary Materials, Table S1). Proteins encoded by these *AhMATE* genes ranged in length from 258 (AhMATE28) amino acids to 582 (AhMATE52) amino acids with an average length of 482 amino acid residues.

EXPASY analysis revealed substantial variations in the molecular weight of AhMATE proteins, ranging from 28.4 kDa (AhMATE28) to 63.9 kDa (AhMATE52), with an average of 52.3 kDa. The predicted isoelectric point (pI) values ranged from 5.38 (AhMATE33, AhMATE89) to 10.03 (AhMATE103), with an average of 7.70. The number of transmembrane segments (TMs) varied from a minimum of six to a maximum of fourteen, with majority AhMATE proteins containing twelve TMs, consistent with their classification as membrane proteins. Detailed characteristics including length, molecular weight, isoelectric point (pI), number of transmembrane segments, and predicted chromosome location of these 111 *AhMATEs*, are provided in the Supplementary Materials, Table S1. Additionally, the multiple sequence alignment of amino acids for several AhMATE proteins is provided in Supplementary Materials, Figure S1.

### 2.2. Phylogenetic Relationship, Conserved Motifs, and Gene Structure Analysis of AhMATEs

**Figure 1:**
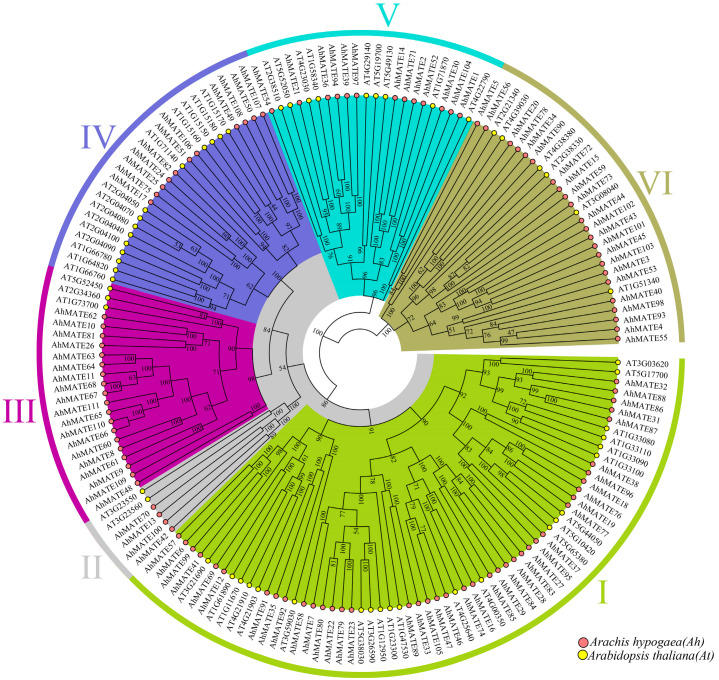
Phylogenetic relationships of MATE genes in peanut and *Arabidopsis*. Tree constructed using the maximum likelihood method. Roman numerals I through VI represent six distinct groups of the MATE gene family, as indicated by different colors. Bootstrap values at the nodes indicate branch confidence. Peanut (*AhMATE*) and *Arabidopsis* (*AtMATE*) members are labeled with pink and yellow solid circles, respectively.

Phylogenetic analysis classified the *AhMATE* genes into six distinct groups (Figure 1). Group I was the largest, comprising forty-one *AhMATE* members clustered with twenty-two *AtMATEs*, followed by Groups VI and IV. In contrast, Group II was the smallest, containing four *AhMATE* genes clustered with two *AtMATEs*, specifically *AT3G23550* and *AT3G23560*. *AhMATE52*, which has a higher molecular weight, was clustered with the *Arabidopsis* gene *AT1G71870* in Group V. *AhMATEs* with the highest number of transmembrane segments, specifically *AhMATE65*, *AhMATE66*, and *AhMATE110*, were clustered together in Group III, alongside three *AtMATE* members: *AT5G52450*, *AT2G34360*, and *AT1G73700*. *AhMATE103*, which has the highest isoelectric point, was closely clustered with *AT1G51340* in Group VI (Figure 1).

The *AhMATE* genes selected for downstream analysis were also identified. *AhMATE77*, *AhMATE95*, *AhMATE83*, *AhMATE29*, and *AhMATE69* were clustered with eight *AtMATE* members in Group I: *AT5G44050/ATDTX28*, *AT5G10420*, *AT5G65380*, *AT4G00350*, *AT1G61890*, *AT1G11670*, *AT4G21910*, and *AT4G21903*. Similarly, in Group IV, *AhMATE106* and *AhMATE49* were clustered with five *AtMATE* members: *AT1G71140*, *AT1G15170*, *AT1G15180*, *AT1G15150*, and *AT1G15160*.

*AhMATE54*, *AhMATE21*, *AhMATE39*, and *AhMATE1* were associated with five *AtMATEs* in Group V: *AT2G38510*, *AT5G52050*, *AT4G23030*, *AT1G58340*, and *AT4G22790*. Additionally, *AhMATE3* and *AhMATE43* were clustered with two *AtMATEs*, *AT1G51340* and *AT3G08040*, in Group VI (Figure 1). The functions of *AhMATE* genes can be inferred from their phylogenetic relationships with known functionally characterized MATE transporters.

**Figure 2:**
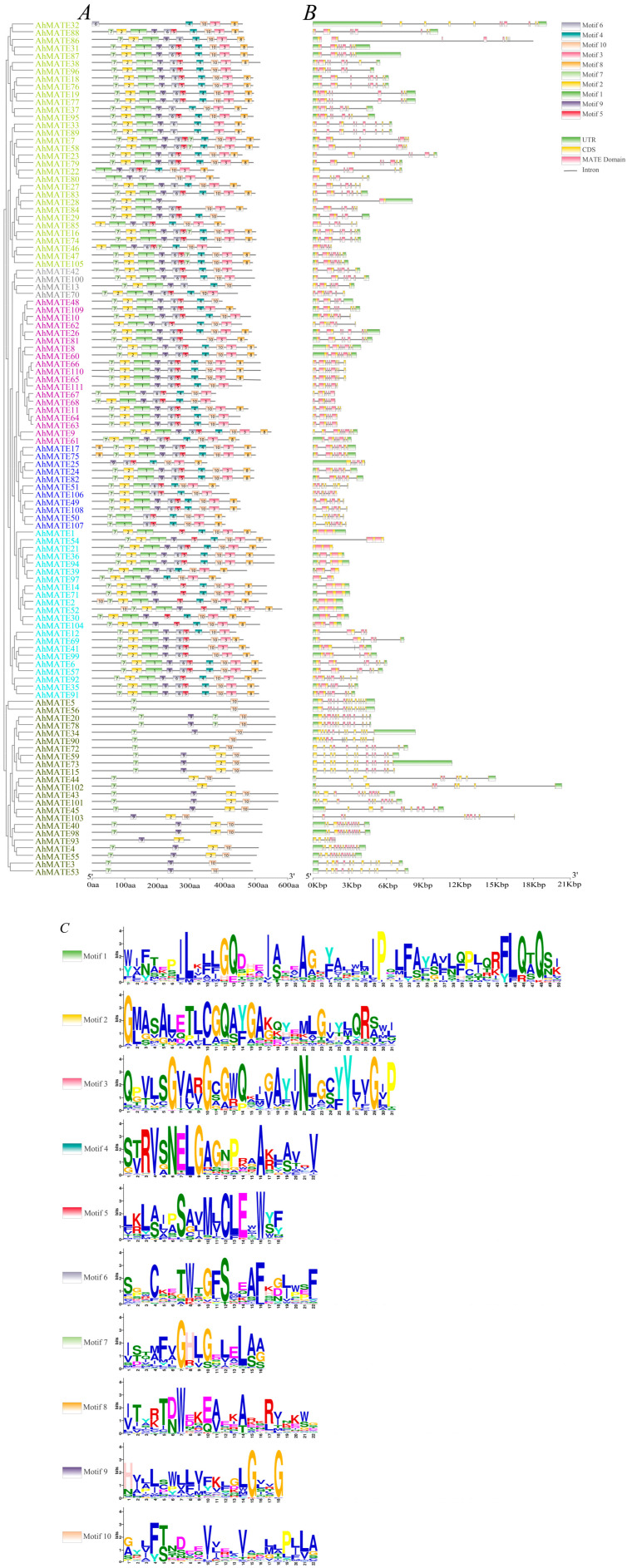
Conserved motifs and gene structure analysis of *AhMATEs*. (**A**) Conserved amino acid motifs number (1–10) in AhMATE proteins are displayed in ten colored boxes, with gray lines indicating amino acid length. (**B**) Gene structure analysis of the *AhMATE* genes. Green rectangles, yellow rectangles, pink rectangles, and gray lines represent UTR (untranslated region), CDS (coding sequence or exons), MATE domain, and introns, respectively (unit: base pair). (**C**) Conserved amino acid motif sequences of AhMATE proteins.

To elucidate the structural characteristics of peanut MATE members, conserved motifs and exon–intron organization were analyzed. The analysis revealed that the number of conserved motifs were consistent within each phylogenetic group, but varied significantly between different groups, aligning with the phylogenetic results. Ten conserved motifs were identified in AhMATE proteins (Figure 2). In Group I, AhMATE32 exhibited a unique motif pattern compared to other members. In Group IV, AhMATE49 contained all ten motifs, whereas AhMATE106 lacked motifs 8 and 9, while both lacked UTR regions. In Group V, most AhMATE proteins contained all ten motifs, except for AhMATE1 and AhMATE54, both of which lacked motif 6 and AhMATE1 protein also lacked motif 9. In Group VI, AhMATE proteins featured motifs 7, 9, and 10, with some members also containing motif 2. Notably, AhMATE3 lacked motif 2, while AhMATE43 retained it. Detailed information regarding the conserved amino acids within these ten motifs can be observed in Figure 2C.

### 2.3. Gene Duplication and Synteny Analysis

**Figure 3:**
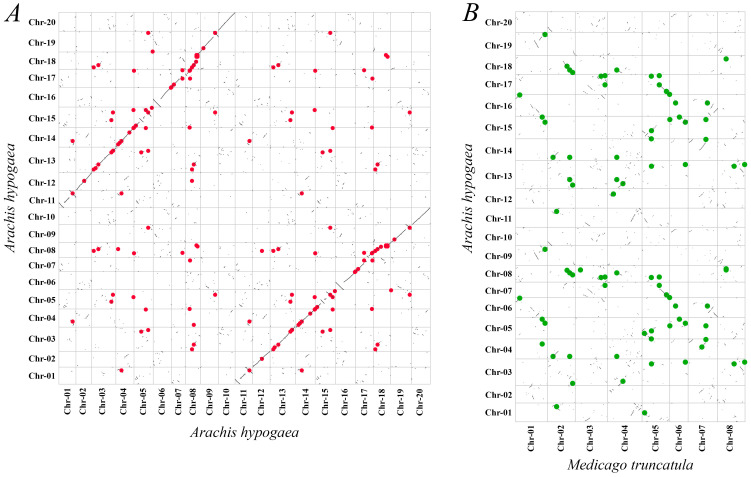
Syntenic analysis of MATE genes in the peanut genome. (**A**) Identification of paralogous MATE gene pairs in micro-syntenic blocks within the *Arachis hypogaea* genome. Paralogous gene pairs are represented by red solid dots. (**B**) Synteny analyses of MATE genes between *Arachis hypogaea* and *Medicago truncatula* genomes. Gene pairs are represented by green solid dots. Dots are plotted according to gene coordination on the respective chromosomes.

The MATE genes in allotetraploid peanut genome were unevenly distributed. Chromosome 5 exhibited the highest density of these loci, with 12% of all *AhMATE* genes annotated to it. In contrast, chromosome 2 and chromosome 11 have the lowest density, with only 1% of *AhMATE* genes annotated to it. Notably, no *AhMATE* genes were mapped to chromosome 10 (Supplementary Materials, Table S1). The selected genes, *AhMATE3*, *AhMATE21*, *AhMATE39*, *AhMATE43*, *AhMATE49*, *AhMATE54*, and *AhMATE106*, were mapped to chromosomes 02, 05, 08, 08, 09, 12, and 19, respectively. Collinearity analysis of paralogous MATE gene pairs within the peanut genome, visualized using a dot plot, revealed structural variations, extensive conserved syntenic blocks, and ten minor inversion breakpoints. Notably, inversions were observed between chr-01 and chr-11, chr-05 and chr-15, chr-06 and chr-16, chr-08 and chr-17, and chr-09 and chr-19 (Figure 3A). In contrast, the dot plot comparison between peanut and *Medicago truncatula* revealed limited conserved synteny, along with numerous minor chromosomal inversions (Figure 3B). Eighty-three gene pairs were identified between these two genomes of peanut and *Medicago truncatula* (Supplementary Materials, Table S2), suggesting high structural variability and a significant evolutionary distance between the two genomes.

To investigate the evolutionary relationships and duplication patterns of MATE genes in peanut, we analyzed syntenic blocks and Ka/Ks ratios of duplicated *AhMATE* gene pairs. The Ka/Ks ratios for all paralogous MATE gene pairs were less than one, indicating that the *AhMATE* genes have undergone purifying selection pressure, maintaining highly conserved structures. Furthermore, two types of gene duplication, mainly segmental and a smaller portion of the tandem duplication, were identified. In total, eighty-four duplicated MATE gene pairs were identified, of which sixty-nine underwent segmental duplication and fifteen underwent tandem duplication (Supplementary Materials, Table S3). *AhMATE* segmental gene pairs were further examined, and their non-synonymous substitution rate, synonymous substitution rate, average N sites, and average S sites were analyzed (Supplementary Materials, Table S4). The selected genes, *AhMATE3*, *AhMATE21*, *AhMATE39*, *AhMATE43*, *AhMATE49*, *AhMATE54*, and *AhMATE106*, all exhibited evidence of purifying selection pressure during evolution. Both segmental and tandem duplication events were observed in these genes (Supplementary Materials, Tables S3 and S4).

### 2.4. Pore Morphology, Dimensions, and Protein Topology

**Figure 4:**
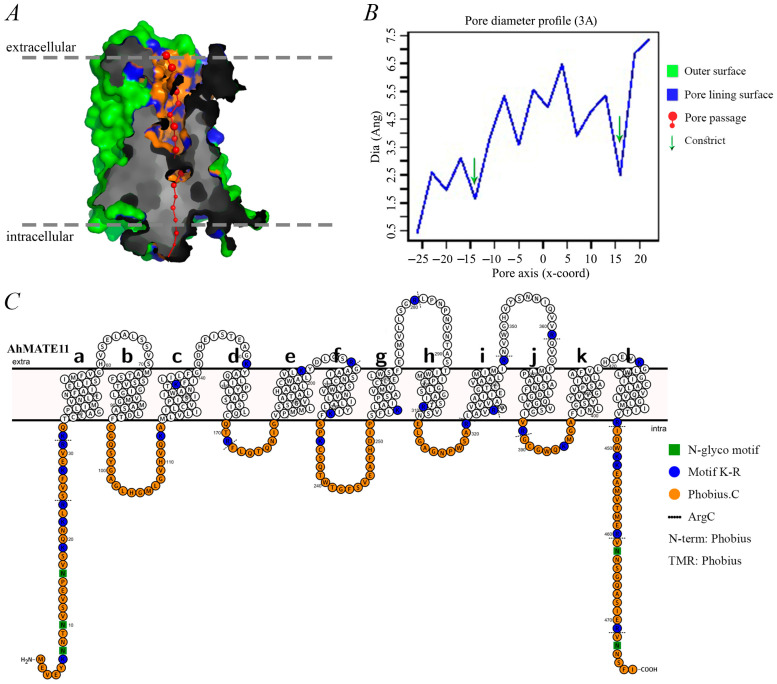
Pore morphology (**A**), dimensions (**B**), and protein topology (**C**) of AhMATE11 protein. (**A**) Protein tertiary structure illustrates the pore morphology characteristics of MATE family members. (**B**) Graph displays pore dimensions obtained from PoreWalker software v1.0. (**C**) Topology of AhMATE11 protein. Letters from a through l represent series of twelve alpha–helical transmembrane regions.

Understanding protein structure is essential for elucidating its function. The AhMATE proteins are characterized as membrane proteins spanning both intracellular and extracellular regions. For instance, the topology of AhMATE11, including its pore morphology and dimensions, is illustrated in Figure 4. PoreWalker v1.0 was used to analyze the pore structure and 3D geometry of all 111 AhMATE proteins, revealing a longitudinally extending pore from the extracellular side to the intracellular side (Figure 4A). Two constrict sites, acting as selectivity barriers, were also observed. Conserved pore sizes and similar constraints were found across all AhMATE proteins, as depicted by green arrows in the pore morphology dimensions diagram (Figure 4B).

Protein topology was used to illustrate the series of twelve alpha–helical transmembrane regions, organized with intramolecular pseudo-two-fold symmetry. These twelve transmembrane segments were arranged into two bundles: TM 1–6 in the N-terminal lobe and TM 7–12 in the C-terminal lobe, forming a pocket with a characteristic V-shaped conformation. A cytoplasmic loop (residues 233–252) between TM6 and TM7 connected the two halves. The initial helix of each half (TM1 and TM7) was preceded by a helical extension (residues 1–33 and 233–252) from the inner membrane leaflet side. An additional helix (residues 447–477) following TM12 was positioned on the cytoplasmic side. The overall structure was predicted to be hourglass-like, with pore-forming amino acids visible within it (Figure 4C).

### 2.5. Cis-Acting Regulatory Elements (CAREs) Detection

**Figure 5:**
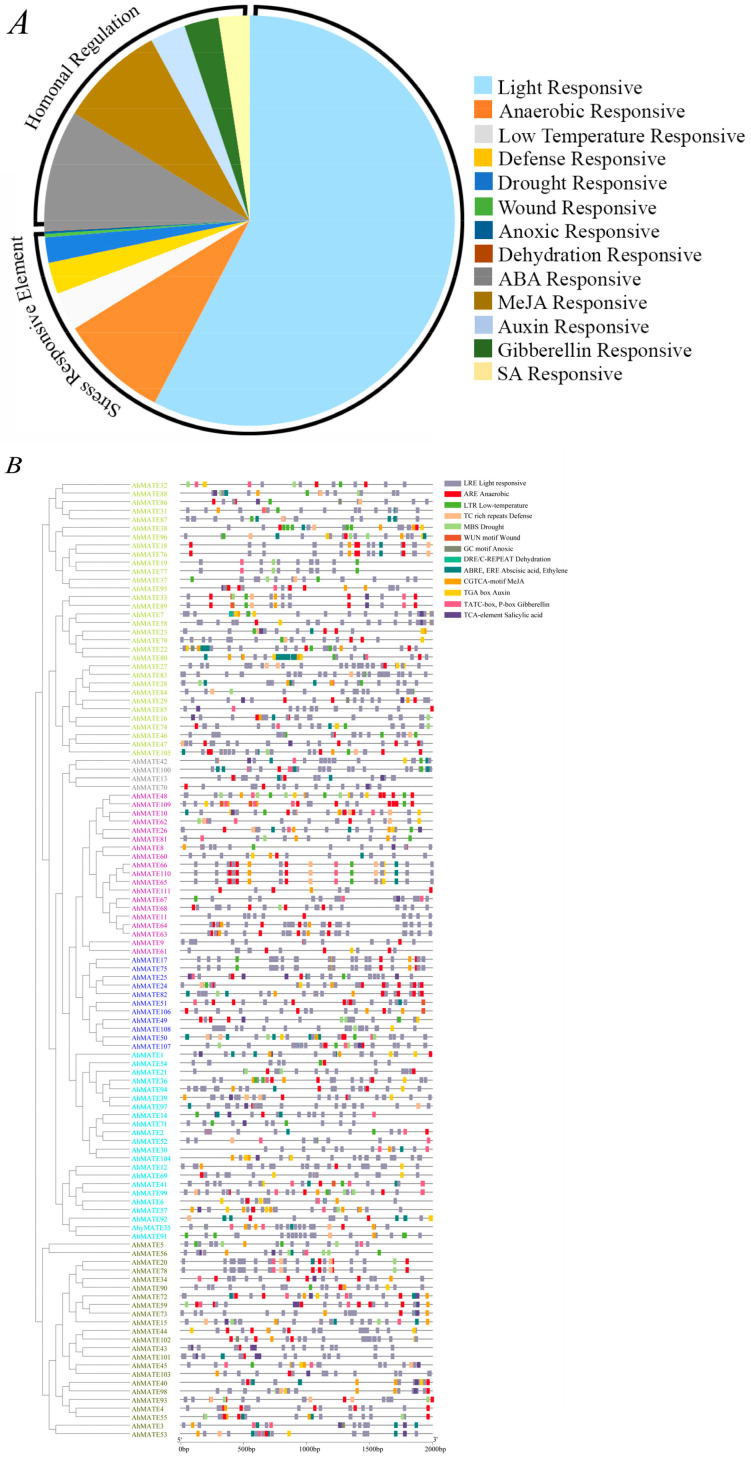
Cis-element analysis of the *AhMATE* genes promoter regions. (**A**) Frequency distribution of stress-responsive and hormonal-responsive cis-elements identified in 2kb promoter sequences of 111 *AhMATE* genes in peanut. (**B**) Schematic representation of stress and hormonal responsive cis-elements composition and localization in *AhMATE* gene promoter regions. Colored rectangles represent different cis-element types and their positions within individual *AhMATE* gene promoter regions.

For growth and stress response, gene transcriptional regulation is vital. To investigate the functional roles of *AhMATE* genes, promoter sequences spanning 2 kb upstream of the translation start site were analyzed. Thirteen distinct types of cis-acting regulatory elements (CAREs) were identified in the promoters of 111 *AhMATE* genes in peanuts. These elements were classified into three functional categories: cellular development, hormonal responsiveness, and stress-responsive elements (Figure 5).

Stress-responsive CAREs were most abundant, with eight types identified at higher frequencies in the *AhMATE* promoter regions: ARE, LTR, TC-rich repeats, MBS, GGNVS motif, WUN motif, GC-motif, and the DRE/C-REPEAT element. These elements are associated with responses to anaerobic conditions, low temperature, defense signaling, drought inducibility, wounding, specific anoxic induction, and dehydration. Additionally, light-responsive elements (LREs) were prevalent across all *AhMATE* promoters, accounting for 58% of the identified CAREs (Figure 5A). Five types of hormonal regulation-responsive cis-elements were identified in *AhMATE* promoters: ABRE (abscisic acid), ERE (ethylene), CGTCA-motif and TGACG-motif (MeJA), TGA-element (auxin), P-box and TATC-box (gibberellin), and TCA-element (salicylic acid) (Figure 5B).

Among the selected *AhMATE* genes, stress-responsive CAREs were annotated as follows: defense-related CAREs were identified in *AhMATE39*, and wound-related CAREs were detected in *AhMATE106*. Phytohormone-related CAREs were identified as follows: abscisic acid and ethylene responsive cis-elements in *AhMATE3*; Methyl jasmonate responsive CAREs in *AhMATE3* and *AhMATE106*; auxin responsive CAREs in *AhMATE39* and *AhMATE49*; gibberellic acid responsive CAREs in *AhMATE3*; salicylic acid responsive CAREs in *AhMATE3*, *AhMATE43*, *AhMATE39*, and *AhMATE49*. These results suggest that the *AhMATE* genes may be involved in diverse stress responses, and plant hormone signaling pathways.

### 2.6. Expression Profiles of AhMATEs Under Al Stress

**Figure 6:**
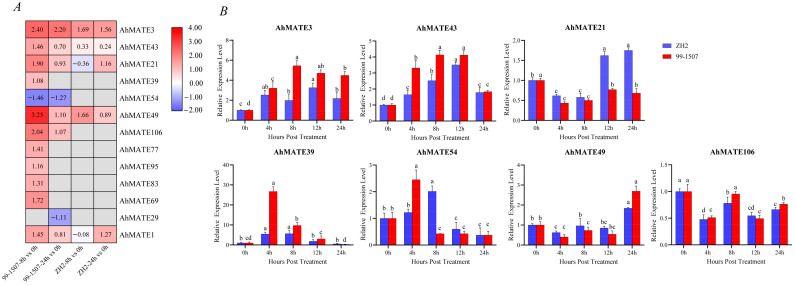
Expression profile of *AhMATE* genes in peanut roots under aluminum stress. (**A**) Heatmap of *AhMATE* genes expression under Al stress. Note: 99-1507-8 h vs 0 h, 99-1507-24 h vs 0 h, ZH2-8 h vs 0 h, and ZH2-24 h vs 0 h denote comparisons of different Al treatment time points in 99-1507 (Al-tolerant) and ZH2 (Al-sensitive) peanut cultivars. The color histogram (right) indicates gene expression level: red (high expression) to dark blue (low expression). Note: A log2-fold change conversion was performed on the expression value. (**B**) Relative expression patterns of seven *AhMATE* genes post-Al treatment time points in 99-1507 and ZH2, quantified by qRT-PCR. The 0 h time point served as the control, with its relative expression set to one. Data were normalized to *UBQ* as an internal reference. Lowercase letters above bars indicate significant differences (*p* < 0.05, Duncan’s test) between different time points.

To elucidate the role of MATE genes in the aluminum (Al) stress response across peanut cultivars, we examined the expression patterns of thirteen *AhMATE* genes predicted to be involved in Al detoxification based on sequence homology and microarray data. Initial RNA-Seq analysis validated the Al stress responsiveness of these thirteen *AhMATE* genes (Figure 6A). Further validation using quantitative real-time PCR (qRT-PCR) focused on seven selected *AhMATE* genes. The ubiquitin (*UBQ*: *FJ438462*) was used as an internal control throughout the experiments.

In Al-tolerant cultivar 99-1507, most *AhMATE* genes exhibited higher expressions at 8 h vs 0 h comparison group under Al stress, though their expression levels declined in the 24 h vs 0 h group. Notably, *AhMATE3*, *AhMATE43*, and *AhMATE49* exhibited higher expression across all comparison groups, with markedly higher levels in Al-tolerant cultivar 99-1507 compared to the Al-sensitive cultivar ZH2. In contrast, *AhMATE21* and *AhMATE1* remained highly expressed in the Al-sensitive cultivar ZH2 at the later stage in the 24 h vs 0 h comparison group (Figure 6A). Relative expression levels of seven *AhMATE* genes in the root tips of two peanut cultivars, analyzed via qRT-PCR, revealed dynamic expression patterns across early (4 h), middle (8 h, 12 h), and late (24 h) time points post-Al treatment. *AhMATE39* and *AhMATE54* exhibited a pronounced up-regulation in expression level at the early stage of the 4 h time point in Al-tolerant cultivar 99-1507 but declined sharply by 24 h in both cultivars. In contrast, *AhMATE49* displayed peak expression at 24 h compared to other time points post-Al treatment (Figure 6B). Notably, *AhMATE21* exhibited uniquely elevated expression in the Al-sensitive cultivar ZH2 during the later stages of 12 h and 24 h post-Al treatment. *AhMATE43* and *AhMATE3* exhibited pronounced up-regulation in both cultivars post-Al treatment, but *AhMATE3* showed relatively higher expression in the Al-tolerant cultivar 99-1507 compared to ZH2. Expressions of both genes peaked during early to middle hours post-Al treatment, indicating early transcriptional responses to Al stress. These expression dynamics suggest the distinct functional roles of these genes under Al stress. *AhMATE43* and *AhMATE3* may likely contribute to Al tolerance mechanisms in the 99-1507 cultivar, while *AhMATE21* may be associated with stress sensitivity in ZH2. 

## 3. Discussion

Gene family analysis has emerged as a crucial approach for elucidating gene structure, evolution, and function in recent years. The MATE family, which plays a vital role in exporting toxins and other substrates, has been widely studied across various plant families including *Poaceae*, *Ericaceae*, *Salicaceae*, and *Solanaceae*. However, relatively little is known about the MATE family in *Fabaceae* [35].

In the present study, 111 *AhMATE* gene members were identified in cultivated peanut. A similar number of MATE gene members have been reported in other related plant species, such as 138 MATE genes in tobacco and 117 MATE genes in soybean [18,36]. In contrast, *Arabidopsis thaliana* has a relatively small MATE family, comprising 56 members [30]. The *AhMATE* encoded proteins ranged from 258 to 582 aa in length and contained 6–14 transmembrane segments (TMs); these results are similar to those of tobacco MATE proteins, which range from 118 to 558 aa and contain 2–14 TMs. Furthermore, soybean MATE proteins have lengths ranging from 80 to 593 aa and contain 2–13 TMs. In comparison, *Arabidopsis* MATE proteins reportedly range from 469 to 575 aa with 12 TMs, reflecting species-specific variability [16]. These findings suggest that MATE family in peanut is characterized by genes that encode proteins of varying lengths, with significant structural diversity among its members.

Phylogenetic analysis reveals evolutionary relationships among groups of organisms and can be used to predict gene function and gene–gene associations [37]. Based on their phylogenetic analysis, the *AhMATE* members were classified into six distinct groups. Among the selected MATE members in Group IV, *AhMATE106* and *AhMATE49* were clustered with five *AtMATE* members, including four MATE efflux proteins (AT1G15170, AT1G15180, AT1G15150, AT1G15160) and one MATE transporter (*AT1G71140/DTX14*). These five *Arabidopsis* MATEs are involved in xenobiotic detoxification and norfloxacin transport [30,38]. In Group V, *AhMATE54*, *AhMATE21*, *AhMATE39*, and *AhMATE1* were associated with five *AtMATEs*, including three MATE efflux proteins (AT4G23030, AT5G52050/DTX50, AT2G38510) and two MATE transporters (*AT4G22790/RHC1*, *AT1G58340/ABS4*) that are known to regulate primary root growth, organ initiation, and CO_2_ signaling [39,40,41]. Additionally, in Group VI, *AhMATE43* and *AhMATE3* clustered with two *AtMATEs*, including one MATE efflux protein (AT3G08040/ATFRD3) that facilitates metal ion transmembrane transporter activity, and a root citrate transporter (*AT1G51340/DTX42*) which contributes to Al tolerance via citrate exudation response [42,43]. The AT3G08040/ATFRD3 ortholog in peanut *AhFRDL1*, has been functionally characterized as mediator of citrate secretion, which contributes in its adaptation to iron deficiency and aluminum stress in peanuts. Given its dual biological functions, *AhFRDL1* may serve as a valuable genetic marker for breeding peanut varieties with high Fe efficiency and Al tolerance [44]. Considering the close phylogenetic relationship between *AhMATEs* and *AtMATEs*, we propose that *AhMATE* members grouped with *AtMATEs* likely share analogous functions, particularly in stress response, CO_2_ signaling, organic acid transport, and the initiation of specific physiological mechanisms.

Introns and exons play a significant role in gene evolution and can influence gene expression [45]. This study revealed that *AhMATEs* predominantly contained ten motifs and exhibit varied exon–intron structures. Closely related *AhMATE* genes within the same group showed similar gene structures, including comparable intron numbers and exon lengths. Motif composition was largely conserved across MATE members in most groups, but Group VI exhibited significant differences, with its members typically containing only three motifs. These results suggest that specific regulatory protein motifs and exon–intron structures may contribute to functional differentiation of various *AhMATE* subfamily genes. Additionally, the preservation of these regulatory elements likely underpins their consistent expression patterns under specific conditions [46].

Sequence analysis revealed highly conserved regions near the transmembrane helices of AhMATE proteins, particularly within their TM linker domains, suggesting that these areas are critical for forming the functional transport pore [47]. Information on pore morphology obtained via PoreWalker v1.0, aids in predicting solute permeability. The orientation of these proteins in the cell membrane may likely facilitates the removal of solutes and other harmful substances, thereby mitigating damage caused by stress conditions [48]. The presence of pore-forming amino acids in MATE proteins enhances their substrate specificity similar to aquaporins, which are known for substrate specificity due to their hydrophobicity and the size of their pore-forming amino acids [49].

The structural topology of MATE transmembranes, distinct from other transporter families like MSF, indicates a unique evolutionary origin and functional adaptation [50]. Notably, structural analyses of AhMATE11, NorM-VC, and hMATE1 (Supplementary Materials, Figure S2) have highlighted a conserved pair of acidic residues E268 in TM7 and D377 in TM10 in the C-lobe, which are crucial for substrate interaction and transport efficiency [12].

Recent crystallographic studies of MATE proteins from *Arabidopsis thaliana* and *Camelina sativa* have highlighted pH-dependent structural changes that are essential for the transport cycle, demonstrating distinct conformations of TM7 under different protonation states [38,51]. These insights support the rocker-switch mechanism proposed for MATE transporters, in which substrate binding and release are regulated by the protonation of key residues within the substrate-binding pocket [52]. Furthermore, the functionality of MATE transporters is evident in their ability to efflux a wide range of substrates. For example, *AtDTX1* not only mediates the efflux of alkaloids such as berberine but also confers tolerance to norfloxacin and cadmium when expressed in *Escherichia coli* [30]. The substrate specificity and transport capacity of MATE proteins are likely influenced by the electrostatic charge distribution within their substrate-binding pockets, a critical factor for selecting and interacting with various ions and molecules [53]. Conserved positions of E268 amino acid residue of TM7 and D377 amino acid residue of TM10 in AhMATE11 (Supplementary Materials, Figure S2), akin to the metal ion binding sites in Vc-NorM, may suggest a comparable functional role in metal ion transport and alkaloid efflux [35].

The *AhMATE* genes exhibited an asymmetrical distribution across all peanut chromosomes. The MATE family in peanuts is notably large, comprising 111 members. Gene duplication has been a key driver in the expansion of this gene family during its evolution [54]. Variations in gene loci likely resulted from gene duplication, gene loss, or chromosomal rearrangements [55]. Duplication analyses revealed two types of gene duplication events in peanut *AhMATE* genes: predominantly by segmental duplication (82%) with a smaller proportion resulting from tandem duplications (18%) (Supplementary Materials, Table S3), supporting the findings of Hanada et al. [56]. These duplication events have a significant role in driving the expansion of the MATE family genes within the peanut genome over the course of its evolutionary history, primarily driven by purifying selection pressure. These events might have facilitated the diversification and functional specialization of MATE family, while purifying selection may has acted to preserve gene functions by eliminating deleterious mutations, thereby maintaining the genomic integrity and adaptive potential of the family [57]. Regarding the limited conserved synteny between peanut and *Medicago truncatula* (Figure 3B, Supplementary Materials, Table S2), it was noted that incorporating synteny with *Medicago truncatula* in the assembly process may obscure structural variations, potentially affecting the observed synteny between two species [58].

Gene expression is regulated by specific cis-acting regulatory elements (CAREs) in the promoter regions, as CAREs are a specific binding site of transcription factors [59]. In this study, phytohormone-related CAREs, including motifs that are responsive to MEJA, ethylene, salicylic acid, and ABA, were identified in various *AhMATEs*: *AhMATE3*, *AhMATE43*, *AhMATE39*, *AhMATE49*, *AhMATE106*. [60]. ABREs (ABA-responsive elements) and EREs (ethylene-responsive elements) were the most frequently observed CAREs in the promoter sequences of these genes. Among other phyto-hormones, the role of ethylene in regulating plant growth under Al-induced growth inhibition is particularly evident [61]. Along with other defense-related motifs, as predicted by NewPLACE v30.0, the GGNVS motif was found at a higher frequency in 2kb upstream promoter regions of these *AhMATE* genes. The GGNVS motif has also been reported in the promoter region of ALUMINUM RESISTANCE TRANSCRIPTION FACTOR 1 (*ART1*) gene in rice. Its higher frequency in the promoter sequence of *AhMATE* genes may suggest a potential role in Al stress defense response. However, the GGNVS motif is short and highly degenerate, its presence alone is insufficient in confirming its functional significance [62].

The expression patterns of genes provide valuable insights into their potential roles. *AhMATE3*, *AhMATE43*, *AhMATE39*, and *AhMATE54* were upregulated early post-Al treatment, while *AhMATE49* and *AhMATE21* expression peaked at later stages in both peanut cultivars. Notably, most of these *AhMATE* genes: *AhMATE3*, *AhMATE43*, *AhMATE39*, *AhMATE54* and *AhMATE49* exhibited greater transcriptional sensitivity to Al treatment in the Al-tolerant cultivar 99-1507 compared to the Al-sensitive cultivar ZH2. Further investigation into the upstream pathways of the aluminum stress response is essential, as the early transcriptional activation of *AhMATE* genes in Al-tolerant cultivar 99-1507 post-Al stress may provide key insights into the mechanism of aluminum tolerance. Expression profiles suggest that these *AhMATE* genes likely play a key role in mitigating Al stress by facilitating organic acid anion transport in peanut roots [63].

Genes within the same group generally exhibited similar expression patterns, with most *AhMATE* gene following this trend except for *AhMATE21*, consistent with gene structure and motif analysis [45]. *AhMATE39* and *AhMATE54*, located on chr-08 and chr-12, were clustered with *AtMATE* transporters involved in CO_2_ signaling and organ initiation. These genes also harbored cis-elements responsive to salicylic acid and indole-3-acetic acid. Notably, they exhibited early transcriptional response in Al-tolerant cultivar 99-1507 post-Al treatment. In contrast, *AhMATE21* located on chr-05, exhibited a delayed response in Al-sensitive cultivar ZH2 post-Al treatment [39]. *AhMATE49* and *AhMATE106*, located on chr-09 and chr-19, respectively, were clustered with an antibiotic transporter, involved in the efflux of endogenous metabolites and xenobiotics, contributing to heavy metal detoxification [30]. *AhMATE49* harbored cis-elements for salicylic acid and indole-3-acetic acid, this gene exhibited higher expression 24 h post-Al treatment. Additionally, *AhMATE3* and *AhMATE43*, located on chr-02 and chr-08, respectively, were clustered with *AtFRD3* and *AtDTX42* transporters, involved in citrate efflux into roots vascular tissue for iron translocation and Al detoxification [64,65]. *AhMATE3* and *AhMATE43* harbored cis-elements responsive to ethylene, MeJA, gibberellic acid, abscisic acid, and salicylic acid, and exhibited relatively higher expression in tolerant cultivar 99-1507 compared to Al-sensitive cultivar ZH2, suggesting their potential role in mitigating Al stress. Collectively, these findings indicate that *AhMATE* genes might play a considerable role in Al-stress response in peanuts. *AhMATE3*, *AhMATE43*, and *AhMATE39*, may likely contribute to Al tolerance mechanisms in the 99-1507 cultivar, while *AhMATE21*, may be associated with stress sensitivity in ZH2.

However, the MATE family exhibits higher flexibility regarding substrate specificity, suggesting that MATE protein-encoded Al resistance in plant species, may not be limited to Al-activated citrate release only. Regulatory loci may be of pivotal importance in fully unlocking the potential for improving Al tolerance based on MATE genes. Therefore, screening the entire MATE family is essential in identifying putative Al-induced citrate transporters from plant species. In summary, this study enhances our understanding of the MATE family’s structural and evolutionary dynamics, and constitutes a foundation for further gene cloning and functional studies.

## 4. Materials and Methods

### 4.1. Identification of MATE Family Genes in Peanut

A hidden Markov model (HMM) profile of the conserved MATE domain PF01554 was downloaded from the pfam database v37.0 that was accessed on 25 April 2024 [66]. MATE proteins in peanut were identified by querying its HHM profile in an HMMER search E-value < 1 × 10^−5^ (http://hmmer.org/) HMMER v3.4 (accessed on 25 April 2024) against the genome sequences of cultivated peanut that were sourced from the PeanutBase Project (https://www.peanutbase.org/download/) (accessed on 25 April 2024). *Arabidopsis thaliana* MATE sequences were downloaded from Phytozome v13 (http://www.phytozome.net/) (accessed on 28 April 2024) with an E-value threshold of <0.01 [67]. Redundant sequences were removed from further analysis based on results generated by ClustalW v2.0 [68]. Additionally, SMART v9.0 and Pfam v37.0 databases were used to verify the presence of MATE domains in the 111 obtained peanut MATE sequences.

The theoretical isoelectric point (pI) and molecular weight (MW) of the peanut MATE proteins were computed using the ExPASy Compute v3.0 pI/Mw tool (https://web.expasy.org/compute_pi/) (accessed on 4 May 2024) [69]. The transmembrane helices numbers were predicted using transporter classification database (http://www.tcdb.org/progs/TMS.php) (accessed on 5 May 2024) and DeepTMHMM Server v1.0 (https://dtu.biolib.com/DeepTMHMM) (accessed on 6 May 2024) [70].

### 4.2. Phylogenetic Tree, Motifs, and Domains Analysis

To understand the evolutionary history of the peanut MATE family in relation to other plants, multiple sequence alignment of 111 peanut MATEs and 56 *Arabidopsis* MATE full-length protein sequences were performed using MUSCLE v5.0 [71]. The trimAl tool v1.5 was used to remove poor-quality alignments [72]. Phylogenetic analysis was performed using IQ-tree v2.3.0 with the maximum likelihood method [73]; node support was assessed with 1000 bootstrap replicates.

The chromosomal locations of the sequences were predicted using Phytozome v13 (https://phytozome.jgi.doe.gov/pz/portal.html) (accessed on 7 June 2024). The peanut MATE protein sequences were compared with those of *Arabidopsis* subfamilies and their conserved domains were analyzed. Motifs in MATE proteins were identified using the Multiple EM for Motif Elicitation v5.5.7 (MEME) (http://meme-suite.org/) (accessed on 11 June 2024) tool with default settings [74]. Gene structures were obtained by comparing genomic sequences and their predicted coding sequences using the gene structure display server v2.0 (https://gsds.gao-lab.org/) (accessed on 14 June 2024) [75].

### 4.3. Duplication Mode and Synteny Analysis

The segmental and tandem duplication events in the peanut MATE family were identified using the Multiple Collinearity Scan toolkit (MCScanX) [76] from the Plant Genome Duplication Database [77] with default settings. Potential anchors between homologous gene pairs were detected using BLASTP (E < 1 × 10^−5^, top 5 matches) and these were subsequently used for MCscan analysis.

The synonymous (Ks) and non-synonymous (Ka) substitution rates for paralogous gene pairs were estimated using SNAP v2.1.1 (https://www.hiv.lanl.gov/content/sequence/SNAP/SNAP.html) (accessed on 5 July 2024). Syntenic blocks were identified with an E-value  ≤  1 × 10^−10^. Tandem duplications were defined as homologous genes on the same chromosome with fewer than ten intervening gene loci and >50% protein similarity [78]. Divergence times were calculated using the formula T = Ks/(2 × 6.5 × 10^−9^) × 10^−6^ Mya [79].

In order to establish strong evidence of homology, microsynteny within the peanut genome was measured, in addition to the syntenic block conservation between the peanut and *Medicago truncatula* genomes (https://data.legumeinfo.org/Medicago/truncatula/genomes/) (accessed on 13 August 2024). Collinear MATE pairs within peanut, and with *Medicago truncatula*, were visualized using the genome dot-plot function in TBtools-II v2.154 [80].

### 4.4. Cis-Acting Regulatory Elements Analysis

The promoter sequences from 2 kb upstream to the translation start site of all MATE genes were retrieved from PeanutBase Project (https://www.peanutbase.org/download/) (accessed on 1 January 2025). The transcriptional responsive elements in *AhMATE* gene promoters were predicted using the New PLACE database v30.0 (https://www.dna.affrc.go.jp/PLACE/?action=newplace) (accessed on 3 January 2025) [81].

### 4.5. Protein Tertiary Structure Prediction

MATE protein sequences were analyzed using Phyre2 v2.2 (http://www.sbg.bio.ic.ac.uk/phyre2) (accessed on 3 October 2024) to model their structures. The resulting protein database files were then submitted to PoreWalker v1.0 (http://www.ebi.ac.uk/thornton-srv/software/PoreWalker/) (accessed on 4 October 2024) to predict their tertiary structures in relation to their pore size. Secondary structural features were further validated by submitting the protein sequences to Protter v1.0 (http://wlab.ethz.ch/protter/) (accessed on 5 October 2024) for the visualization of proteoforms and the integration of annotated sequence features with experimental proteomic evidence.

### 4.6. Expression Analysis of AhMATEs

The peanut root tip transcriptome data (PRJNA525247) from our previous study were used to analyze the expression patterns of *AhMATEs* under Al stress. The heatmap was visualized using TBtools-II software v2.154.

### 4.7. Plant Growth, Al Treatment, and RNA Isolation

The seeds of two peanut cultivars, 99-1507 (Al-tolerant) and ZH2 (Al-sensitive), were sourced from the Oil Crops Research Institute of Chinese Academy of Agricultural Science (Hubei, China). Peanut plants were routinely planted on farm of Guangxi University in Nanning, Guangxi Province, China (22°53′06.7″ N 108°21′36.6″ E). The seeds of the two selected cultivars were germinated in moist, sterile perlite under dark conditions at 26 °C in a growth chamber. Seedlings with roots measuring approximately three cm in length were transferred to 1/5 Hoagland nutrient solution and grown under a 14 h day/10 h night photoperiod at 26/24 °C (day/night) for three days. Subsequently, the seedlings were transferred to 0.1 mM CaCl_2_ (pH 4.3) for 24 h prior to Al treatment. The seedlings were exposed to 0.1 mM CaCl_2_ (pH 4.3) solution with either 0 μM AlCl_3_ (control) or 100 μM AlCl_3_ (treatment) for 4, 8, 12, and 24 h, respectively. The root tips (0–1 cm) were collected, immediately frozen in liquid nitrogen, and stored at − 80 °C. The experiment was performed in triplicate. Total RNA was extracted from all samples using TRIzol Reagent (Invitrogen, Carlsbad, CA, USA), following the manufacturer’s protocol.

### 4.8. Quantitative Real-Time PCR

Gene-specific primers were designed using primer premier 5.0 (Premier Biosoft International, Palo Alto, CA, USA) and synthesized by Invitrogen (Shanghai, China). Quantitative real-time PCR was performed on a Roche 480 Real-time Detection System (Roche Diagnostics, Basel, Switzerland) following the manufacturer’s instructions. The qRT-PCR reaction volume was 15 μL, containing 2 μL cDNA, 7.5 μL 2× SYBR Premix Ex Taq (TaKaRa, Kyoto, Japan), and 50 nM each of forward and reverse primers. The amplification program consisted of initial denaturation at 95 °C for 5 min followed by 40 cycles of denaturation at 95 °C for 10 s, annealing at 58 °C for 20 s, and extension at 72 °C for 20 s. The amplification efficiencies (E) of primer pairs for eight genes, including the housekeeping gene *UBQ*, were estimated using quantitative real-time PCR using 1×, 5×, 10×, 20×, and 30× dilutions of cDNA, according to the following equation: E = [10^(−1/slope)^] − 1 [82].

The detailed quantitative real-time PCR primer sequences are provided in the Supplementary Materials, Table S5. The relative expression values were calculated using the 2^−ΔΔCT^ method according to Livak and Schmittgen [83], with *UBQ* as an internal control. The relative expression levels of *AhMATE* genes in response to Al stress (100 μM AlCl_3_) were compared to control conditions (0 μM AlCl_3_) at each time point.

### 4.9. Statistical Analysis

The experiments were independently replicated three times and their mean values were subjected to data processing and statistical analysis using Microsoft Excel 2016 and IBM SPSS statistics v26.0 (IBM Corp., Armonk, NY, USA). Values were presented as the mean ± standard deviation (SD) of three biological replications. Bar graphs were generated using GraphPad Prism v9.0 (GraphPad software, LLC, San Diego, CA, USA), with error bars representing standard deviation from three measurements. The significance test was performed using two-way ANOVA followed by Duncan’s post hoc test. The letters above the bars indicate significant differences (*p* < 0.05, Duncan’s test) between different time points.

## 5. Conclusions

This study identified and characterized 111 *AhMATE* genes in cultivated peanuts. Based on their domain architectures and evolutionary relationships, these genes were classified into six distinct groups. Peanut *AhMATE* genes have underwent purifying selection pressure and experienced segmental and tandem duplication events. Promoter analysis revealed that the majority of *AhMATE* genes contain hormone-responsive and stress-responsive cis-elements. Furthermore, expression analyses of seven *AhMATE* gene post-Al treatment time points, showed that these genes can respond to Al stress and may have different expression levels in two peanut cultivars. Taken together, these findings provide a comprehensive characterization of the *AhMATE* gene family in cultivated peanuts.

## Data Availability

We state that all data necessary for confirming the conclusions presented in the article are represented fully within the article.

## Supplementary Materials

The supporting information can be downloaded at: www.mdpi.com/article/10.3390/ijms26062707/s1.

## Abbreviations

The following abbreviations are used in this manuscript:

Al	aluminum
aa	amino acids
bp	base pair
CARE	cis-acting regulatory element
CDS	coding sequence
TMs	transmembrane segments
UTR	untranslated region
ZH2	Zhonghua No.2
