# Supplementary material for: Genome-Wide Dissection of MATE Gene Family in Cultivated Peanuts and Unveiling Their Expression Profiles Under Aluminum Stress
Source: Int J Mol Sci. 2025 Mar 17;26(6):2707. doi: 10.3390/ijms26062707 (PMC11942301; doi:10.3390/ijms26062707)
Supplement: Supplementary file 1 [file ijms-26-02707-s001.zip › Supp_Material_Supp_Figure_IJMS_3435638.pdf]

Table S1. Characteristics of MATE family genes in peanut (*Arachis hypogaea* L.)**.**

Gene Name	Gene ID	Size	MW(kDa)	pI	TMs	Chr	Start	End	Strand
AhMATE1	Ahy-arahy.Tifrunner. gnm2.ann1.2QIE74.1	503	54.7	8.42	12	1	47260162	47262824	+
AhMATE2	Ahy-arahy.Tifrunner. gnm2.ann1.22RBTX.1	510	55.5	6.70	11	1	101506563	101508992	+
AhMATE3	Ahy-arahy.Tifrunner. gnm2.ann1.BV54DM.1	485	52.6	6.59	11	2	62443831	62451136	−
AhMATE4	Ahy-arahy.Tifrunner. gnm2.ann1.N01QY3.1	510	54.5	8.55	12	3	3343216	3347508	−
AhMATE5	Ahy-arahy.Tifrunner. gnm2.ann1.IPZ39I.1	542	58.2	8.98	11	3	6609203	6614240	+
AhMATE6	Ahy-arahy.Tifrunner. gnm2.ann1.IBJJ46.1	521	56.3	5.49	12	3	13942249	13948273	+
AhMATE7	Ahy-arahy.Tifrunner. gnm2.ann1.T29US9.1	514	56.1	7.55	12	3	132507356	132515168	−
AhMATE8	Ahy-arahy.Tifrunner. gnm2.ann1.4S5LXH.1	504	54.4	6.21	12	3	136583292	136587198	+
AhMATE9	Ahy-arahy.Tifrunner. gnm2.ann1.UQFX5N.1	549	59.9	9.15	12	3	136608815	136612432	+
AhMATE10	Ahy-arahy.Tifrunner. gnm2.ann1.X8YVGZ.2	486	53.5	8.68	10	3	136614032	136617089	+
AhMATE11	Ahy-arahy.Tifrunner. gnm2.ann1.N8KRVL.1	478	51.8	8.87	12	3	136949579	136951853	+
AhMATE12	Ahy-arahy.Tifrunner. gnm2.ann1.8D4CC7.1	441	47.5	5.66	10	4	4668478	4672863	−
AhMATE13	Ahy-arahy.Tifrunner. gnm2.ann1.PBN9WF.1	486	53.6	6.67	10	4	8891361	8894724	+
AhMATE14	Ahy-arahy.Tifrunner. gnm2.ann1.V3YXUH.1	535	58.3	7.87	12	4	17492147	17495095	+
AhMATE15	Ahy-arahy.Tifrunner. gnm2.ann1.C41B5E.1	553	59.2	6.46	11	4	102992594	102999254	+

AhMATE16	Ahy-arahy.Tifrunner. gnm2.ann1.ITY3SF.1	502	54.5	5.86	12	4	117418086	117421916	−
AhMATE17	Ahy-arahy.Tifrunner. gnm2.ann1.B3W5YN.1	501	53.9	5.92	12	4	127817981	127821458	+
AhMATE18	Ahy-arahy.Tifrunner. gnm2.ann1.VXI9Y2.1	493	53.3	6.39	12	5	26488	32650	−
AhMATE19	Ahy-arahy.Tifrunner. gnm2.ann1.SM4FP5.1	495	54.5	6.75	12	5	33603	41957	−
AhMATE20	Ahy-arahy.Tifrunner. gnm2.ann1.W8BY14.2	562	60.2	9.00	10	5	3998021	4002737	−
AhMATE21	Ahy-arahy.Tifrunner. gnm2.ann1.KLG2UC.1	535	57.9	8.80	12	5	12737918	12739522	−
AhMATE22	Ahy-arahy.Tifrunner. gnm2.ann1.Y6ZMDV.1	373	40.5	6.37	9	5	34557548	34564796	+
AhMATE23	Ahy-arahy.Tifrunner. gnm2.ann1.08HZBK.1	460	50	8.00	11	5	34794050	34804158	+
AhMATE24	Ahy-arahy.Tifrunner. gnm2.ann1.2H67VJ.1	496	53.7	8.16	12	5	91207854	91211439	+
AhMATE25	Ahy-arahy.Tifrunner. gnm2.ann1.65MVIR.1	352	38.5	8.35	10	5	91286452	91290686	+
AhMATE26	Ahy-arahy.Tifrunner. gnm2.ann1.WMKD0W.1	489	53	6.70	12	5	101051800	101057247	+
AhMATE27	Ahy-arahy.Tifrunner. gnm2.ann1.P7VUGU.1	455	50.1	8.32	11	5	113390454	113394324	−
AhMATE28	Ahy-arahy.Tifrunner. gnm2.ann1.KJPV2Q.1	258	28.4	8.22	6	5	113404179	113412290	−
AhMATE29	Ahy-arahy.Tifrunner. gnm2.ann1.14330B.1	407	44.9	6.37	10	5	113432895	113437492	−
AhMATE30	Ahy-arahy.Tifrunner. gnm2.ann1.09KEBU.1	485	52.7	8.61	12	5	115164688	115167597	+
AhMATE31	Ahy-arahy.Tifrunner. gnm2.ann1.S65VS3.1	494	54.3	7.61	12	6	87302607	87307247	−
AhMATE32	Ahy-arahy.Tifrunner.	461	50.5	9.80	6	6	87520143	87539193	−

	gnm2.ann1.W2E3A5.1								
AhMATE33	Ahy-arahy.Tifrunner.gnm2.ann1.15LFE4.1	468	51	5.38	12	6	117883116	117889545	–
AhMATE34	Ahy-arahy.Tifrunner.gnm2.ann1.S9LP7J.1	552	59.2	9.28	11	7	284038	292388	–
AhMATE35	Ahy-arahy.Tifrunner.gnm2.ann1.FWRT0A.2	511	55.6	5.98	12	7	4043766	4047434	–
AhMATE36	Ahy-arahy.Tifrunner.gnm2.ann1.2WQJ7Q.1	559	61.2	7.50	11	7	67498117	67500647	–
AhMATE37	Ahy-arahy.Tifrunner.gnm2.ann1.WPG9ZU.1	475	52.1	5.75	12	8	13679483	13684357	–
AhMATE38	Ahy-arahy.Tifrunner.gnm2.ann1.CU8EVV.1	515	56.4	6.71	12	8	13691770	13697229	–
AhMATE39	Ahy-arahy.Tifrunner.gnm2.ann1.B4CBXE.1	474	51.2	7.10	10	8	15705041	15707151	–
AhMATE40	Ahy-arahy.Tifrunner.gnm2.ann1.37C7XW.1	521	55.6	7.67	12	8	26927017	26931611	+
AhMATE41	Ahy-arahy.Tifrunner.gnm2.ann1.B845YX.1	482	52	6.08	10	8	31949577	31954335	+
AhMATE42	Ahy-arahy.Tifrunner.gnm2.ann1.IK0U2W.1	490	53.8	6.69	12	8	39831632	39835461	–
AhMATE43	Ahy-arahy.Tifrunner.gnm2.ann1.Z17CL9.1	570	60.6	9.29	12	8	41053668	41060341	+
AhMATE44	Ahy-arahy.Tifrunner.gnm2.ann1.ZAC0X2.1	422	45.2	9.56	9	8	41069237	41084132	+
AhMATE45	Ahy-arahy.Tifrunner.gnm2.ann1.YHN8YH.1	538	58.6	9.29	8	8	44588364	44599014	–
AhMATE46	Ahy-Manual_mRNA_1	396	44.4	9.01	9	9	5874374	5875882	–
AhMATE47	Ahy-arahy.Tifrunner.gnm2.ann1.VI8BIT.1	501	55.1	8.35	12	9	5882017	5884709	–
AhMATE48	Ahy-arahy.Tifrunner.gnm2.ann1.1L1WJ8.1	399	43.7	5.70	9	9	112767151	112770406	–
AhMATE49	Ahy-Manual_mRNA_9	454	49.8	8.65	11	9	112790312	112792825	+

AhMATE50	Ahy-Manual_mRNA_10	408	44.1	6.88	10	9	112793869	112796377	+
AhMATE51	Ahy-Manual_mRNA_11	389	41.7	6.87	8	9	112797310	112800170	+
AhMATE52	Ahy-arahy.Tifrunner.<br>gnm2.ann1.HSA1AU.1	582	63.9	8.40	11	11	141746825	141749265	−
AhMATE53	Ahy-arahy.Tifrunner.<br>gnm2.ann1.DH6IGP.1	492	53.4	7.18	11	12	85509143	85516916	−
AhMATE54	Ahy-arahy.Tifrunner.<br>gnm2.ann1.RHB0JA.1	548	60.2	8.30	11	12	108332112	108337889	+
AhMATE55	Ahy-arahy.Tifrunner.<br>gnm2.ann1.ZRA95S.1	504	53.7	8.55	12	13	5601540	5605495	−
AhMATE56	Ahy-arahy.Tifrunner.<br>gnm2.ann1.1DJ7XZ.1	542	58.2	9.08	11	13	7922896	7927922	−
AhMATE57	Ahy-arahy.Tifrunner.<br>gnm2.ann1.WG9HC3.1	521	56.4	5.65	12	13	17850191	17855872	+
AhMATE58	Ahy-arahy.Tifrunner.<br>gnm2.ann1.FJ2DZD.1	511	55.6	7.52	12	13	135014215	135021866	−
AhMATE59	Ahy-arahy.Tifrunner.<br>gnm2.ann1.Z9FA5A.1	543	57.9	8.14	11	13	138728493	138735497	−
AhMATE60	Ahy-arahy.Tifrunner.<br>gnm2.ann1.HS29P7.1	504	54.5	6.15	12	13	138827201	138830745	+
AhMATE61	Ahy-arahy.Tifrunner.<br>gnm2.ann1.Q5QJ3U.1	451	49.1	9.33	12	13	138859094	138862214	+
AhMATE62	Ahy-arahy.Tifrunner.<br>gnm2.ann1.3BU2IH.2	459	51	8.91	10	13	138863615	138867094	+
AhMATE63	Ahy-arahy.Tifrunner.<br>gnm2.ann1.8W4GAU.1	454	49.5	9.11	11	13	139174962	139176936	+
AhMATE64	Ahy-arahy.Tifrunner.<br>gnm2.ann1.ZY7B9V.1	454	49.5	9.11	11	13	139207439	139209414	+
AhMATE65	Ahy-arahy.Tifrunner.<br>gnm2.ann1.JFC2EZ.1	516	56.1	9.26	14	14	3024489	3027161	+
AhMATE66	Ahy-arahy.Tifrunner.<br>gnm2.ann1.09ED9P.1	516	56.1	9.18	14	14	3083392	3086057	+
AhMATE67	Ahy-arahy.Tifrunner.	379	41.8	8.04	10	14	3247258	3249031	−

	gnm2.ann1.GX8W1W.1								
AhMATE68	Ahy-arahy.Tifrunner. gnm2.ann1.CTT4B3.1	377	41.1	8.11	11	14	3430980	3432783	—
AhMATE69	Ahy-arahy.Tifrunner. gnm2.ann1.747ND8.1	463	50.2	7.53	10	14	5937389	5944818	—
AhMATE70	Ahy-arahy.Tifrunner. gnm2.ann1.EN8HK1.1	446	48.8	8.66	11	14	10273467	10276060	+
AhMATE71	Ahy-arahy.Tifrunner. gnm2.ann1.DBIL6B.1	536	58.5	7.47	12	14	17903134	17906137	+
AhMATE72	Ahy-arahy.Tifrunner. gnm2.ann1.1ERV7Y.2	491	52.9	8.87	9	14	112841676	112849414	+
AhMATE73	Ahy-arahy.Tifrunner. gnm2.ann1.TND3PH.1	543	58.1	8.19	12	14	112895843	112907188	+
AhMATE74	Ahy-arahy.Tifrunner. gnm2.ann1.SGPK7M.1	503	54.7	5.51	12	14	130173741	130177628	—
AhMATE75	Ahy-arahy.Tifrunner. gnm2.ann1.Y8F5KD.1	501	53.9	5.92	12	14	142253511	142256988	+
AhMATE76	Ahy-arahy.Tifrunner. gnm2.ann1.3HHC30.1	493	53.3	6.39	12	15	26488	32650	—
AhMATE77	Ahy-arahy.Tifrunner. gnm2.ann1.BBQR41.1	495	54.6	6.75	12	15	33603	41957	—
AhMATE78	Ahy-arahy.Tifrunner. gnm2.ann1.J04VDZ.2	562	60.2	9.00	10	15	3998021	4002737	—
AhMATE79	Ahy-arahy.Tifrunner. gnm2.ann1.M8SMER.1	494	54.1	8.55	12	15	22485600	22492871	—
AhMATE80	Ahy-arahy.Tifrunner. gnm2.ann1.UAS7KI.1	390	42.4	6.30	9	15	22547831	22552420	—
AhMATE81	Ahy-arahy.Tifrunner. gnm2.ann1.K7F148.1	476	51.8	6.45	12	15	143105604	143110435	—
AhMATE82	Ahy-arahy.Tifrunner. gnm2.ann1.77VLFZ.1	496	53.6	8.34	12	15	153468961	153473052	—
AhMATE83	Ahy-arahy.Tifrunner. gnm2.ann1.3S3RK6.1	500	55.3	8.43	12	15	158414763	158419213	—

AhMATE84	Ahy-Manual_mRNA_6	474	52.6	6.92	12	15	158423000	158426609	—
AhMATE85	Ahy-Manual_mRNA_7	407	45.2	8.39	11	15	158427822	158431408	—
AhMATE86	Ahy-arahy.Tifrunner.gnm2.ann1.K8VWBY.1	467	51.9	9.18	11	16	115865966	115882027	—
AhMATE87	Ahy-arahy.Tifrunner.gnm2.ann1.E7UEEQ.1	494	54.3	7.63	12	16	116064043	116071199	—
AhMATE88	Ahy-arahy.Tifrunner.gnm2.ann1.D0WG0L.1	463	51.1	9.17	11	16	116314581	116324771	—
AhMATE89	Ahy-arahy.Tifrunner.gnm2.ann1.ZK1PB2.1	468	51	5.38	12	16	150150075	150156504	—
AhMATE90	Ahy-arahy.Tifrunner.gnm2.ann1.GK4T5X.1	532	57	9.20	10	17	443478	448444	—
AhMATE91	Ahy-arahy.Tifrunner.gnm2.ann1.716UDI.2	511	55.5	6.22	12	17	4768836	4772250	—
AhMATE92	Ahy-Manual_mRNA_15	532	57.9	8.75	12	17	4780200	4783830	—
AhMATE93	Ahy-arahy.Tifrunner.gnm2.ann1.H64M7Z.1	300	31.8	6.04	6	17	8152099	8153916	+
AhMATE94	Ahy-arahy.Tifrunner.gnm2.ann1.QQ9WEM.1	558	61	7.50	10	17	34881332	34884281	+
AhMATE95	Ahy-arahy.Tifrunner.gnm2.ann1.G80ETX.1	494	54.1	5.99	12	17	129770360	129775395	—
AhMATE96	Ahy-arahy.Tifrunner.gnm2.ann1.6P2K74.1	458	49.6	7.03	12	17	129788345	129793312	—
AhMATE97	Ahy-arahy.Tifrunner.gnm2.ann1.NFLV1C.1	394	42.2	8.77	10	17	131645222	131646897	—
AhMATE98	Ahy-arahy.Tifrunner.gnm2.ann1.1Y45Q7.1	521	55.6	7.67	12	18	3044268	3048927	+
AhMATE99	Ahy-arahy.Tifrunner.gnm2.ann1.SGT6RC.1	495	53.4	6.25	12	18	8068299	8073490	+
AhMATE100	Ahy-arahy.Tifrunner.gnm2.ann1.3YRJ0Q.1	498	54.8	6.49	12	18	25641569	25646132	—
AhMATE101	Ahy-arahy.Tifrunner.gnm2.ann1.BK8UPU.1	570	60.6	9.35	12	18	114793631	114800880	+

AhMATE102	Ahy-arahy.Tifrunner.gnm2.ann1.5DIS84.1	438	47.3	9.38	9	18	114823699	114844001	+
AhMATE103	Ahy-arahy.Tifrunner.gnm2.ann1.PWI1R1.1	370	40.7	10.03	9	18	124786240	124802688	−
AhMATE104	Ahy-arahy.Tifrunner.gnm2.ann1.7NDL57.1	514	56	8.15	11	19	662353	664651	−
AhMATE105	Ahy-arahy.Tifrunner.gnm2.ann1.4U2MDP.1	492	54	8.51	12	19	8143164	8146034	−
AhMATE106	Ahy-Manual_mRNA_4	420	44.7	7.84	9	19	156317755	156319690	−
AhMATE107	Ahy-Manual_mRNA_12	408	44	7.50	10	19	156320980	156323683	−
AhMATE108	Ahy-Manual_mRNA_14	455	49.9	8.68	11	19	156324873	156327661	−
AhMATE109	Ahy-arahy.Tifrunner.gnm2.ann1.K8SE68.1	440	47.8	5.76	10	19	156351675	156355497	+
AhMATE110	Ahy-arahy.Tifrunner.gnm2.ann1.C634CF.1	516	56.1	9.26	14	scaffold_66	19319	21989	+
AhMATE111	Ahy-arahy.Tifrunner.gnm2.ann1.Q5L44J.1	459	49.9	8.80	12	scaffold_66	46556	48558	+

MW: molecular weight, pI: isoelectric point, TMs: transmembrane segments, Chr: Chromosome.

Table S2. Synteny analyses between peanut and *Medicago truncatula*.

Pair	Sequence 1	Sequence 2
Pair_1	*AhMATE2*	Medtr2g435930.1.JCVIMt4.0v1
Pair_2	*AhMATE1*	Medtr5g011540.1.JCVIMt4.0v1
Pair_3	*AhMATE4*	Medtr2g097900.1.JCVIMt4.0v1
Pair_4	*AhMATE5*	Medtr4g071370.1.JCVIMt4.0v1
Pair_5	*AhMATE7*	Medtr5g033100.1.JCVIMt4.0v1
Pair_6	*AhMATE8*	Medtr6g081410.1.JCVIMt4.0v1
Pair_7	*AhMATE8*	Medtr8g106670.1.JCVIMt4.0v1
Pair_8	*AhMATE7*	Medtr8g069470.1.JCVIMt4.0v1
Pair_9	*AhMATE16*	Medtr1g100180.1.JCVIMt4.0v1
Pair_10	*AhMATE12*	Medtr2g022980.1.JCVIMt4.0v1
Pair_11	*AhMATE12*	Medtr2g088400.1.JCVIMt4.0v1
Pair_12	*AhMATE12*	Medtr4g048000.1.JCVIMt4.0v1
Pair_13	*AhMATE15*	Medtr7g070210.1.JCVIMt4.0v1
Pair_14	*AhMATE17*	Medtr7g082800.1.JCVIMt4.0v1
Pair_15	*AhMATE27*	Medtr1g100180.1.JCVIMt4.0v1
Pair_16	*AhMATE26*	Medtr1g108810.1.JCVIMt4.0v1
Pair_17	*AhMATE21*	Medtr5g010830.1.JCVIMt4.0v1
Pair_18	*AhMATE18*	Medtr5g032720.1.JCVIMt4.0v1
Pair_19	*AhMATE22*	Medtr5g033100.1.JCVIMt4.0v1
Pair_20	*AhMATE24*	Medtr6g004220.2.JCVIMt4.0v1
Pair_21	*AhMATE27*	Medtr6g047590.1.JCVIMt4.0v1
Pair_22	*AhMATE26*	Medtr6g081410.1.JCVIMt4.0v1
Pair_23	*AhMATE24*	Medtr7g082800.1.JCVIMt4.0v1
Pair_24	*AhMATE33*	Medtr1g021740.1.JCVIMt4.0v1
Pair_25	*AhMATE31*	Medtr6g027200.1.JCVIMt4.0v1
Pair_26	*AhMATE31*	Medtr7g087370.1.JCVIMt4.0v1
Pair_27	*AhMATE36*	Medtr3g111020.1.JCVIMt4.0v1
Pair_28	*AhMATE36*	Medtr5g067460.1.JCVIMt4.0v1
Pair_29	*AhMATE34*	Medtr5g099020.1.JCVIMt4.0v1
Pair_30	*AhMATE35*	Medtr5g090280.1.JCVIMt4.0v1
Pair_31	*AhMATE41*	Medtr2g088400.1.JCVIMt4.0v1
Pair_32	*AhMATE40*	Medtr2g097900.1.JCVIMt4.0v1
Pair_33	*AhMATE42*	Medtr2g078930.1.JCVIMt4.0v1
Pair_34	*AhMATE37*	Medtr3g099700.1.JCVIMt4.0v1
Pair_35	*AhMATE43*	Medtr3g029510.2.JCVIMt4.0v1
Pair_36	*AhMATE39*	Medtr3g111020.1.JCVIMt4.0v1
Pair_37	*AhMATE41*	Medtr4g048000.1.JCVIMt4.0v1
Pair_38	*AhMATE39*	Medtr5g067460.1.JCVIMt4.0v1
Pair_39	*AhMATE37*	Medtr5g032720.1.JCVIMt4.0v1
Pair_40	*AhMATE43*	Medtr8g037170.1.JCVIMt4.0v1
Pair_41	*AhMATE45*	Medtr8g037170.1.JCVIMt4.0v1
Pair_42	*AhMATE48*	Medtr1g108810.1.JCVIMt4.0v1

Pair_43	*AhMATE49*	Medtr1g108840.1.JCVIMt4.0v1
Pair_44	*AhMATE52*	Medtr2g435930.1.JCVIMt4.0v1
Pair_45	*AhMATE54*	Medtr4g027920.1.JCVIMt4.0v1
Pair_46	*AhMATE57*	Medtr2g088400.1.JCVIMt4.0v1
Pair_47	*AhMATE55*	Medtr2g097900.1.JCVIMt4.0v1
Pair_48	*AhMATE56*	Medtr4g071370.1.JCVIMt4.0v1
Pair_49	*AhMATE57*	Medtr4g048000.1.JCVIMt4.0v1
Pair_50	*AhMATE58*	Medtr5g033100.1.JCVIMt4.0v1
Pair_51	*AhMATE60*	Medtr6g081410.1.JCVIMt4.0v1
Pair_52	*AhMATE60*	Medtr8g106670.1.JCVIMt4.0v1
Pair_53	*AhMATE58*	Medtr8g069470.1.JCVIMt4.0v1
Pair_54	*AhMATE69*	Medtr2g022980.1.JCVIMt4.0v1
Pair_55	*AhMATE69*	Medtr2g088400.1.JCVIMt4.0v1
Pair_56	*AhMATE69*	Medtr4g048000.1.JCVIMt4.0v1
Pair_57	*AhMATE75*	Medtr7g082800.1.JCVIMt4.0v1
Pair_58	*AhMATE81*	Medtr1g108810.1.JCVIMt4.0v1
Pair_59	*AhMATE83*	Medtr1g100180.1.JCVIMt4.0v1
Pair_60	*AhMATE79*	Medtr5g033100.1.JCVIMt4.0v1
Pair_61	*AhMATE76*	Medtr5g032720.1.JCVIMt4.0v1
Pair_62	*AhMATE81*	Medtr6g081410.1.JCVIMt4.0v1
Pair_63	*AhMATE83*	Medtr6g047590.1.JCVIMt4.0v1
Pair_64	*AhMATE82*	Medtr6g004220.2.JCVIMt4.0v1
Pair_65	*AhMATE82*	Medtr7g082800.1.JCVIMt4.0v1
Pair_66	*AhMATE89*	Medtr1g021740.1.JCVIMt4.0v1
Pair_67	*AhMATE86*	Medtr6g027200.1.JCVIMt4.0v1
Pair_68	*AhMATE86*	Medtr7g087370.1.JCVIMt4.0v1
Pair_69	*AhMATE95*	Medtr3g099700.1.JCVIMt4.0v1
Pair_70	*AhMATE94*	Medtr3g111020.1.JCVIMt4.0v1
Pair_71	*AhMATE97*	Medtr3g111020.1.JCVIMt4.0v1
Pair_72	*AhMATE97*	Medtr5g067460.1.JCVIMt4.0v1
Pair_73	*AhMATE91*	Medtr5g090280.1.JCVIMt4.0v1
Pair_74	*AhMATE90*	Medtr5g099020.1.JCVIMt4.0v1
Pair_75	*AhMATE94*	Medtr5g067460.1.JCVIMt4.0v1
Pair_76	*AhMATE95*	Medtr5g032720.1.JCVIMt4.0v1
Pair_77	*AhMATE99*	Medtr2g088400.1.JCVIMt4.0v1
Pair_78	*AhMATE98*	Medtr2g097900.1.JCVIMt4.0v1
Pair_79	*AhMATE100*	Medtr2g078930.1.JCVIMt4.0v1
Pair_80	*AhMATE99*	Medtr4g048000.1.JCVIMt4.0v1
Pair_81	*AhMATE103*	Medtr8g037170.1.JCVIMt4.0v1
Pair_82	*AhMATE106*	Medtr1g108840.1.JCVIMt4.0v1
Pair_83	*AhMATE109*	Medtr1g108810.1.JCVIMt4.0v1

Table S3. Duplication mode and divergence time of AhMATE duplicated pairs in peanut.

Sequence 1	Sequence 2	Ka	Ks	Ka/Ks	Mode of Duplication	Selection Type	Divergence Time (Mya)
AhMATE2	*AhMATE14*	0.194	1.509	0.128	Segmental	Purifying	123.702
AhMATE2	*AhMATE52*	0.009	0.030	0.293	Segmental	Purifying	2.452
AhMATE2	*AhMATE71*	0.192	1.565	0.123	Segmental	Purifying	128.280
AhMATE3	*AhMATE53*	0.007	0.044	0.167	Segmental	Purifying	3.593
AhMATE8	*AhMATE26*	0.194	0.741	0.261	Segmental	Purifying	60.743
AhMATE7	*AhMATE22*	0.173	1.159	0.150	Segmental	Purifying	94.979
AhMATE4	*AhMATE40*	0.137	0.655	0.209	Segmental	Purifying	53.654
AhMATE7	*AhMATE58*	0.009	0.055	0.165	Segmental	Purifying	4.481
AhMATE8	*AhMATE60*	0.014	0.048	0.293	Segmental	Purifying	3.947
AhMATE11	*AhMATE63*	0.054	0.100	0.544	Segmental	Purifying	8.170
AhMATE4	*AhMATE55*	0.006	0.021	0.291	Segmental	Purifying	1.755
AhMATE5	*AhMATE56*	0.002	0.013	0.195	Segmental	Purifying	1.032
AhMATE7	*AhMATE79*	0.169	1.225	0.138	Segmental	Purifying	100.376
AhMATE8	*AhMATE81*	0.208	0.780	0.267	Segmental	Purifying	63.920
AhMATE4	*AhMATE98*	0.137	0.653	0.209	Segmental	Purifying	53.505
AhMATE17	*AhMATE24*	0.161	0.765	0.211	Segmental	Purifying	62.670
AhMATE12	*AhMATE41*	0.270	1.368	0.197	Segmental	Purifying	112.161
AhMATE14	*AhMATE52*	0.183	1.543	0.118	Segmental	Purifying	126.487

AhMATE16	*AhMATE74*	0.007	0.031	0.223	Segmental	Purifying	2.575
AhMATE12	*AhMATE69*	0.022	0.064	0.340	Segmental	Purifying	5.278
AhMATE13	*AhMATE70*	0.017	0.018	0.959	Segmental	Purifying	1.465
AhMATE14	*AhMATE71*	0.007	0.034	0.222	Segmental	Purifying	2.768
AhMATE17	*AhMATE82*	0.160	0.754	0.213	Segmental	Purifying	61.786
AhMATE19	*AhMATE37*	0.240	1.497	0.161	Segmental	Purifying	122.671
AhMATE26	*AhMATE48*	0.320	2.068	0.155	Segmental	Purifying	169.514
AhMATE26	*AhMATE60*	0.186	0.730	0.255	Segmental	Purifying	59.813
AhMATE22	*AhMATE58*	0.180	1.126	0.160	Segmental	Purifying	92.330
AhMATE24	*AhMATE75*	0.161	0.765	0.211	Segmental	Purifying	62.670
AhMATE27	*AhMATE83*	0.008	0.046	0.169	Segmental	Purifying	3.740
AhMATE24	*AhMATE82*	0.004	0.028	0.128	Segmental	Purifying	2.286
AhMATE26	*AhMATE81*	0.007	0.030	0.252	Segmental	Purifying	2.425
AhMATE18	*AhMATE95*	0.343	1.727	0.199	Segmental	Purifying	141.533
AhMATE26	*AhMATE109*	0.317	1.856	0.171	Segmental	Purifying	152.145
AhMATE30	*AhMATE104*	0.004	0.043	0.085	Segmental	Purifying	3.523
AhMATE36	*AhMATE39*	0.219	1.795	0.122	Segmental	Purifying	147.110
AhMATE35	*AhMATE91*	0.006	0.024	0.248	Segmental	Purifying	2.007
AhMATE36	*AhMATE97*	0.175	2.143	0.082	Segmental	Purifying	175.638
AhMATE34	*AhMATE90*	0.022	0.056	0.399	Segmental	Purifying	4.608

AhMATE36	*AhMATE94*	0.003	0.054	0.058	Segmental	Purifying	4.457
AhMATE43	*AhMATE45*	0.250	0.868	0.288	Segmental	Purifying	71.160
AhMATE40	*AhMATE53*	0.268	2.185	0.123	Segmental	Purifying	179.121
AhMATE41	*AhMATE57*	0.129	1.033	0.124	Segmental	Purifying	84.673
AhMATE40	*AhMATE55*	0.133	0.671	0.199	Segmental	Purifying	54.976
AhMATE37	*AhMATE76*	0.354	1.742	0.203	Segmental	Purifying	142.819
AhMATE38	*AhMATE77*	0.343	1.954	0.176	Segmental	Purifying	160.150
AhMATE37	*AhMATE95*	0.006	0.049	0.131	Segmental	Purifying	4.045
AhMATE39	*AhMATE97*	0.006	0.047	0.122	Segmental	Purifying	3.823
AhMATE39	*AhMATE94*	0.217	1.768	0.123	Segmental	Purifying	144.930
AhMATE40	*AhMATE98*	0.003	0.031	0.083	Segmental	Purifying	2.553
AhMATE41	*AhMATE99*	0.002	0.039	0.048	Segmental	Purifying	3.187
AhMATE43	*AhMATE101*	0.007	0.039	0.177	Segmental	Purifying	3.237
AhMATE45	*AhMATE103*	0.046	0.092	0.499	Segmental	Purifying	7.540
AhMATE42	*AhMATE100*	0.013	0.029	0.439	Segmental	Purifying	2.368
AhMATE45	*AhMATE101*	0.244	0.856	0.285	Segmental	Purifying	70.164
AhMATE43	*AhMATE103*	0.232	0.837	0.277	Segmental	Purifying	68.625
AhMATE48	*AhMATE81*	0.334	1.999	0.167	Segmental	Purifying	163.875
AhMATE46	*AhMATE105*	0.046	0.101	0.459	Segmental	Purifying	8.249
AhMATE48	*AhMATE109*	0.055	0.126	0.435	Segmental	Purifying	10.302

AhMATE49	*AhMATE106*	0.339	1.338	0.253	Segmental	Purifying	109.669
AhMATE52	*AhMATE71*	0.182	1.583	0.115	Segmental	Purifying	129.733
AhMATE58	*AhMATE79*	0.169	1.134	0.149	Segmental	Purifying	92.961
AhMATE60	*AhMATE81*	0.197	0.748	0.263	Segmental	Purifying	61.334
AhMATE57	*AhMATE99*	0.123	1.026	0.119	Segmental	Purifying	84.094
AhMATE55	*AhMATE98*	0.134	0.666	0.201	Segmental	Purifying	54.558
AhMATE75	*AhMATE82*	0.160	0.754	0.213	Segmental	Purifying	61.786
AhMATE76	*AhMATE95*	0.343	1.727	0.199	Segmental	Purifying	141.533
AhMATE81	*AhMATE109*	0.361	2.075	0.174	Segmental	Purifying	170.090
AhMATE94	*AhMATE97*	0.182	1.958	0.093	Segmental	Purifying	160.519
AhMATE101	*AhMATE103*	0.232	0.874	0.265	Segmental	Purifying	71.614
AhMATE49	*AhMATE50*	0.153	0.546	0.280	Tandem	Purifying	44.767
AhMATE106	*AhMATE107*	0.379	1.288	0.294	Tandem	Purifying	105.534
AhMATE91	*AhMATE92*	0.031	0.122	0.253	Tandem	Purifying	10.025
AhMATE84	*AhMATE85*	0.169	0.405	0.418	Tandem	Purifying	33.186
AhMATE83	*AhMATE84*	0.182	0.477	0.383	Tandem	Purifying	39.059
AhMATE22	*AhMATE23*	0.157	0.638	0.246	Tandem	Purifying	52.295
AhMATE76	*AhMATE77*	0.271	1.257	0.216	Tandem	Purifying	102.996
AhMATE8	*AhMATE9*	0.381	1.538	0.247	Tandem	Purifying	126.068
AhMATE101	*AhMATE102*	0.265	0.768	0.345	Tandem	Purifying	62.959

AhMATE95	*AhMATE96*	0.367	1.625	0.226	Tandem	Purifying	133.221
AhMATE60	*AhMATE61*	0.326	1.448	0.225	Tandem	Purifying	118.656
AhMATE27	*AhMATE28*	0.217	0.566	0.383	Tandem	Purifying	46.362
AhMATE79	*AhMATE80*	0.184	0.720	0.256	Tandem	Purifying	59.014
AhMATE18	*AhMATE19*	0.271	1.257	0.216	Tandem	Purifying	102.996
AhMATE43	*AhMATE44*	0.263	0.747	0.352	Tandem	Purifying	61.193

Ka: non-synonymous substitutions, Ks: synonymous substitutions, Mya: million years ago.

Table S4. Analysis of AhMATE segmental duplication pairs and their Ka Ks value.

Sequence 1	Sequence 2	Ka	Ks	Ka/Ks	Effective Len	Average S-sites	Average N-sites
AhMATE2	*AhMATE14*	0.194	1.509	0.128	1515	372.333	1142.667
AhMATE2	*AhMATE52*	0.009	0.030	0.293	1524	375.167	1148.833
AhMATE2	*AhMATE71*	0.192	1.565	0.123	1515	373.583	1141.417
AhMATE3	*AhMATE53*	0.007	0.044	0.167	1452	352.333	1099.667
AhMATE8	*AhMATE26*	0.194	0.741	0.261	1458	353.667	1104.333
AhMATE7	*AhMATE22*	0.173	1.159	0.150	1116	267.083	848.917
AhMATE4	*AhMATE40*	0.137	0.655	0.209	1527	382.833	1144.167
AhMATE7	*AhMATE58*	0.009	0.055	0.165	1524	373.000	1151.000
AhMATE8	*AhMATE60*	0.014	0.048	0.293	1509	364.500	1144.500
AhMATE11	*AhMATE63*	0.054	0.100	0.544	1335	321.417	1013.583
AhMATE4	*AhMATE55*	0.006	0.021	0.291	1509	379.000	1130.000
AhMATE5	*AhMATE56*	0.002	0.013	0.195	1623	400.500	1222.500
AhMATE7	*AhMATE79*	0.169	1.225	0.138	1464	356.417	1107.583
AhMATE8	*AhMATE81*	0.208	0.780	0.267	1419	343.750	1075.250
AhMATE4	*AhMATE98*	0.137	0.653	0.209	1527	383.500	1143.500
AhMATE17	*AhMATE24*	0.161	0.765	0.211	1452	360.000	1092.000
AhMATE12	*AhMATE41*	0.270	1.368	0.197	1305	336.500	968.500
AhMATE14	*AhMATE52*	0.183	1.543	0.118	1512	373.500	1138.500
AhMATE16	*AhMATE74*	0.007	0.031	0.223	1503	357.583	1145.417
AhMATE12	*AhMATE69*	0.022	0.064	0.340	1320	336.250	983.750
AhMATE13	*AhMATE70*	0.017	0.018	0.959	1227	297.250	929.750
AhMATE14	*AhMATE71*	0.007	0.034	0.222	1602	393.750	1208.250
AhMATE17	*AhMATE82*	0.160	0.754	0.213	1449	358.583	1090.417
AhMATE19	*AhMATE37*	0.240	1.497	0.161	1374	326.500	1047.500
AhMATE26	*AhMATE48*	0.320	2.068	0.155	1182	283.667	898.333
AhMATE26	*AhMATE60*	0.186	0.730	0.255	1458	353.500	1104.500

AhMATE22	*AhMATE58*	0.180	1.126	0.160	1116	268.167	847.833
AhMATE24	*AhMATE75*	0.161	0.765	0.211	1452	360.000	1092.000
AhMATE27	*AhMATE83*	0.008	0.046	0.169	1362	316.250	1045.750
AhMATE24	*AhMATE82*	0.004	0.028	0.128	1485	365.333	1119.667
AhMATE26	*AhMATE81*	0.007	0.030	0.252	1425	344.750	1080.250
AhMATE18	*AhMATE95*	0.343	1.727	0.199	1449	347.667	1101.333
AhMATE26	*AhMATE109*	0.317	1.856	0.171	1305	314.833	990.167
AhMATE30	*AhMATE104*	0.004	0.043	0.085	1452	359.083	1092.917
AhMATE36	*AhMATE39*	0.219	1.795	0.122	1422	339.333	1082.667
AhMATE35	*AhMATE91*	0.006	0.024	0.248	1530	373.667	1156.333
AhMATE36	*AhMATE97*	0.175	2.143	0.082	1182	287.750	894.250
AhMATE34	*AhMATE90*	0.022	0.056	0.399	1587	403.083	1183.917
AhMATE36	*AhMATE94*	0.003	0.054	0.058	1671	400.333	1270.667
AhMATE43	*AhMATE45*	0.250	0.868	0.288	1560	378.667	1181.333
AhMATE40	*AhMATE53*	0.268	2.185	0.123	1413	350.583	1062.417
AhMATE41	*AhMATE57*	0.129	1.033	0.124	1440	367.917	1072.083
AhMATE40	*AhMATE55*	0.133	0.671	0.199	1509	379.333	1129.667
AhMATE37	*AhMATE76*	0.354	1.742	0.203	1392	332.333	1059.667
AhMATE38	*AhMATE77*	0.343	1.954	0.176	1440	344.333	1095.667
AhMATE37	*AhMATE95*	0.006	0.049	0.131	1422	335.000	1087.000
AhMATE39	*AhMATE97*	0.006	0.047	0.122	1173	287.500	885.500
AhMATE39	*AhMATE94*	0.217	1.768	0.123	1422	339.833	1082.167
AhMATE40	*AhMATE98*	0.003	0.031	0.083	1560	393.333	1166.667
AhMATE41	*AhMATE99*	0.002	0.039	0.048	1440	369.500	1070.500
AhMATE43	*AhMATE101*	0.007	0.039	0.177	1707	415.917	1291.083
AhMATE45	*AhMATE103*	0.046	0.092	0.499	1038	257.000	781.000
AhMATE42	*AhMATE100*	0.013	0.029	0.439	1467	352.917	1114.083
AhMATE45	*AhMATE101*	0.244	0.856	0.285	1560	378.583	1181.417

AhMATE43	*AhMATE103*	0.232	0.837	0.277	1107	275.750	831.250
AhMATE48	*AhMATE81*	0.334	1.999	0.167	1143	273.583	869.417
AhMATE46	*AhMATE105*	0.046	0.101	0.459	1164	280.500	883.500
AhMATE48	*AhMATE109*	0.055	0.126	0.435	1164	279.417	884.583
AhMATE49	*AhMATE106*	0.339	1.338	0.253	1170	287.250	882.750
AhMATE52	*AhMATE71*	0.182	1.583	0.115	1512	374.500	1137.500
AhMATE58	*AhMATE79*	0.169	1.134	0.149	1464	356.750	1107.250
AhMATE60	*AhMATE81*	0.197	0.748	0.263	1419	343.750	1075.250
AhMATE57	*AhMATE99*	0.123	1.026	0.119	1479	376.250	1102.750
AhMATE55	*AhMATE98*	0.134	0.666	0.201	1509	380.000	1129.000
AhMATE75	*AhMATE82*	0.160	0.754	0.213	1449	358.583	1090.417
AhMATE76	*AhMATE95*	0.343	1.727	0.199	1449	347.667	1101.333
AhMATE81	*AhMATE109*	0.361	2.075	0.174	1305	315.500	989.500
AhMATE94	*AhMATE97*	0.182	1.958	0.093	1182	288.167	893.833
AhMATE101	*AhMATE103*	0.232	0.874	0.265	1107	275.333	831.667

Ka: non-synonymous substitutions, Ks: synonymous substitutions.

Table S5. Seven Peanut MATE gene-specific primers used for qRT-PCR analysis.

Gene Name	Gene ID	Sequence
AhMATE3	LOC107474651	M3-F:CGAGGAAGATGCCGTCGATA<br>M3-R:TCCTCGGTCACAGAAGAGGT
AhMATE43	LOC107462830	M43-F:GTGGCCCTCTCTCTTGTTGT<br>M43-R:CGGGATGCCAACTCTGATGA
AhMATE21	LOC107488068	M21-F:CATACACCGCGCTTCTTTCG<br>M21-R:GCCTAACGCGATTCCTCTGA
AhMATE39	LOC107461007	M39-F:GCACAGAGCATTACGCTTCC<br>M39-R:ACAAGGAGGAAGTTCAGCGG
AhMATE54	LOC107475610	M54-F:GCGGTTTTGCACCTACCAAT<br>M54-R:TGGAATTCAATCCGGTGGCT
AhMATE49	LOC107467460	M49-F:TGGTGATGGCAGCAATGAGT<br>M49-R:CATATCCAAAGATGTGGCGGC
AhMATE106	LOC107467430	M106-F:GTTTTGTGTGGCCAAGACCC<br>M106-R:CAAGCGAAAATGCTGCTCCT
UBQ	UBQ10R	F:CGCACACTCGCTGACTACAAC<br>R:CACGGAGACGGAGGACAAGG

Figure S1. Amino acid multiple sequence alignment of several AhMATEs.

```
                                                                        TM1                                      TM2                                     TM3
AhMATE75            -----MNKEEAAIPLLLTRREEEEKEVNKNSNNNNINGVVESALFWQELKRVGSMAAPMVAVTVSQYLLQVVSMMMVGHLGVLVTFSGVAIATSFAEVTGFSILIGMAGALETLCGQTYGAEEFRSLGNYTCCAIGTLTLVCLPISLIWI     145
AhMATE81            ------------MEREEHQKASLHSPLVQDWGEQDKTKSIEKSEVLEEVKKQLWLSGPLVSVSLLNYCLQVISVMFVGHLGELPLSGSASMATSFASVTGFSLLVGMASALDTLCGQSYGAKQHRMLGIHMQRAMLILMIVSVPLAVIWI     138
AhMATE26            ------------MEREEHQKASLHSPLIQDCAEQNKAKSIEKSEVVEEVKKQLWLSGPLVSVSLLNYCLQVISVMFVGHLGELALSG-ASMATSFASVTGFSLLVGMASALDTLCGQSYGAKQHRMLGIHMQRAMLILMIVSVPLAVIWI     137
AhMATE42            MLGSKNKSCHGSAATTTPLLLGDREEVEDAQ----HEEWWKRILDMDEAKHQLLFSLPMIVTNLLYYLITLVSVMLVGHLGDLQLAG-STLANSWFSVTGVSLMVGLSGALETLCGQGFGAKEYQMLGIYLQSSCIISFVFSIIISIIWF     146
AhMATE100           MLGSNNKSSHGTAATTTPLLLGDREEVEDAQQQEQHEEWWKRILDMDEAKHQLLFSLPMIVTNLLYYLITLVSVMLVGHLGDLQLAG-STLANSWFSVTGVSLMVGLSGALETLCGQGFGAKEYQMLGIYLQSSCIISFVFSIIISIIWF     149
AhMATE19            --MPNKENSALVEAKVPLLNSNQSLRVHENGDDQNHEDDVLSQRIWIESKKLWHIVGPAIFSRITSYMILVITQAFAGHLGDLELAA-FSISNNVIVGFDFGLLLGMASALETLCGQAYGAKKHYMLGVYMQRSWIVLFICCIFLVPLYV     147
Clustal Consensus                                   .                          * ::   :  * :   :  : : :::  :.**:. *   .  :::..     ...:::*::.**:*:*** :**  .  ** :   :      : .: :  ::

                         TM4                      TM5                      TM6                                  TM7
AhMATE75            FMDKILLLFGQDPAISLVAREYCICLIPALYGYAVLQAMTRYFQTQSMIYPMVFSSVAALCFHVPICWGLVFKFGLGHIGAAFAIAISYWLNAIGLVVYMKCSSSCEKSKIVFSSNALKCISEFLQYAIPSGLMFCFEWWSFELLTLFSG     295
AhMATE81            NTRSILMFLGQDPEISTEAGSYAQLMVPSLFAYGLLQCLNRFLQTQNIVFPMVFCSGITTLLHVLICWIMVFKSGLGSRGAAIANSISYWINVLLLSLYVKLSPSCARTWSGFSKEAFQNIPSFLRLAIPSAVMVCLEMWSFEMMVLLSG     288
AhMATE26            NTRSILMFLGQDPEISTEAGSYAQLMVPSLFAYGLLQCLNRFLQTQNIVFPMVFCSGITTLLHVLICWIMVFKSGLGSRGAAIANSISYWINVLLLSLYVKFSPSCARTWSGFSKEAFQNIPSFLRLAIPSAVMVCLEMWSFEMMVLLSG     287
AhMATE42            YTQHILVLLHQSQDIAKTAALYMKFFFPGLFAYGFLQNILRFLQTQSVVTPLVILSAIPLLIHIGIAYVLVEKTGLGFVGAPISASISLWIAIVLLALYVIYGKNFKLTWEGFSTQSFHYVLPTLKLALPSAAMVCLEYWAFEVLVFLAG     296
AhMATE100           YTQHILVLLHQSQDISKTAALYMKFFFPGLFAYGFLQNILRFLQTQSVVTPLVILSAIPLLIHIGIAYTLVEKTGLGFVGAPISASISLWIAIVLLALYVMYGKNFKLTWEGFSTQSFHYVFPSLKLALPSAAMVCLEYWAFEVLVFLAG     300
AhMATE19            FATPVLRVLGQPEELAVLAGEVALWMIPLHFSFAFQFPLQRFLQSQLKTAVIAWVSLVSLLVHIAISWFFVSQLKFGVVGTTATVNFSWWVMTLGLFGYTVSG-GCPNTWTGFSLEAFAGLWDFVKLSASSGLMLCLENWYYRILILMTG     297
Clustal Consensus       :* .: *   ::  *       :.*  :. ..     *::*:*      :   *  .  .*  :.: :* :  :*  *:. :  :* *:  : *  *     .       :   ** :::  :   :: : .*. *.*:* * :.:: :::*

                                TM8                        TM9                                 TM10                       TM11
AhMATE75            LLPNPQLETSILSVCLNTTTLHFFIPYAVGASASTRVSNELGAGNPKAAKGIIRVIVTVAVTEAVIVSSFFICFRNVLGNAYSNDMQVIENVAKMAPLLCVSVIADSLIGVLSGVARGGGFQQIGAYVNLGGYYLVGAPIALFLGFGLKL     445
AhMATE81            LLPNPQLETSVLSICLNTAAAVWMIPFGLS-------------GAVRTARLAVCVVLVMAIIEGLIVGLVLILIRHVWGYAYSNEGEVVDYVAVMLPILATSNFLDGLQCVLSGTARGCGWQKIGAYVNLGSYYLVGIPSAILLAFTFHI     425
AhMATE26            LLPNPQLETSVLSICLNTAAAVWMIPFGLSGAVSTRVSNELGAGNPRTARLAVCVVLVMAIIEGLIVGLVLILIRHVWGYAYSNEGEVVDYVAVMLPILATSNFLDGLQCVLSGTARGCGWQKIGAYVNLGSYYLVGIPSAILLAFTFHI     437
AhMATE42            IMPDSEITTSLIAICANTEFIAYMITYGLSAAASTRVSNELGAGHPDRANHAMRVTLTLSVLLGLCFVLALVFGHNIWIQMFSSSSKIKEEFASITPLLAISILLDAVQGVLS----GCGRQHLAAYVNLATFYLIGLPISCLLGFKTNL     442
AhMATE100           IMPDSEITTSLIAICANTEFIAYMITYGLSAAASTRVSNELGAGHPDQAIRAMRVTLTLSVLLGLCFVLALVFGHNIWIQMFSSSSKIKEEFASITPLLAISILLDAVQGVLSGVIRGCGRQHLAAYVNLVTFYLIGLPISCLLGFKTNL     450
AhMATE19            NLPNAEIAVDALSICMTINGLEMMIPLAFFAATGVRVANELGAGNGKGAKFATKVSVMTSIAIGLFFWMLILIFHDKFGYIFSTSKPVLDEVNKLSLLLAFTILLNSVQPVLSGVAVGSGWQSYVAYINLGCYYLIGVPLGLLMGWVFNQ     447
Clustal Consensus    :*:.           .      :*. ..              *    *      * :  ::  .  .   :: :.     :*..  :   .  :  :*. : : :.:  *:*     * * *   *::**  :*::* * . .:.:  :

                          TM12
AhMATE75            NAKGLWMGTLSG-SCLVVIILAIVTALTDWEKEATKARERIAENSIKDGDVSVSSV-      501
AhMATE81            GGKGLWLGIICA-LIVQVLSLLTITIRTDWDQEAKKATDRVFDSITPDSIVS-----      476
AhMATE26            GGKGLWLGIICAGLIVQVLSLLTITIRTDWDQEAKKATDRVFDSITPDSIVS-----      489
AhMATE42            QFKGLWIGLICG-LVCQSGALLFFTWRANWSKLHLSLDKIRHNDQPIVV--------      490
AhMATE100           QFKGLWIGLICG-LVCQTGALLFFTWRANWSKLHLSLDKIRHNDQPIVV--------      498
AhMATE19            GVMGVWAGMIFGGTATQTLILGLITIRCDWDKEAERAKLHLRKWDNPK---------      495
Clustal Consensus      *:* * : .        *  .*   :*.:          .
```

Figure S1. Multiple sequence alignment of several AhMATEs amino acid sequences was performed using ClustalW v2.0 with default settings. Black bars above the alignments indicate twelve potential transmembrane domains (TM1–TM12). Identical residues are marked with asterisks (*), highly conserved residues with colons (:), and less conserved residues with single dots (.). Gaps in the alignment are represented by dashes (–).

Figure S2. Amino acid sequence analysis of NorM-VC, hMATE1 and AhMATE11.

```
                                                                  TM1                                TM2
NorM-VC         -----------------------MENSVHRYKKEASNLIKLATPVLIASVAQTGMGFVDTIMAGGVSAIDMAAVSIAASIW-LPSILFGVGLLMALVPVV 76
hMATE1          MEAPEEPAPVRGGPEATLEVRGSRCLRLSAFREELRALLVLAGPAFLVQLMVFLISFISSVFCGHLGKLELDAVTLAIAVINVTGVSVGFGLSSACDTLI 100
AhMATE11        -----MEVEYKNNTNVSVEPNVSKQNKLRSVFKEVRKQTCLAGPLIMVNLLNFAIELISIMFVGHVSELALSSVSMATSFSSVTGIGLVMGMASALDTFC 95
                                                 *      ** *                   *         *  *             *   *

                                TM3                                TM4                      TM5
NorM-VC         AQLNGAGRQHKIPFEVHQGLILALLVSVPIIAVLFQTQFIIRFMDVEEAMATKTVGYMHAVIFAVPAYLLFQALRSFTDGMSLTKPAMVIGFIGLLLNIP 176
hMATE1          SQTYGSQNLKHVGVILQRSALVLLLCCFPCWALFLNTQHILLLFRQDPDVSRLTQTYVTIFIPALPATFLYMLQVKYLLNQGIVLPQIVTGVAANLVNAL 200
AhMATE11        GQSYGAGLHGMLGVHVQKAMLVLIILCVPISIIWANTKFILILFGQDHEISTEAGKYAQLIIPYLFASSILQCQTRFLQTQNIGVPMMLSSGVATALHAA 195
                 *  *                       *       *  *                *    *    *                  *

                          TM6                                                     TM7
NorM-VC         LNWIFVYGKFGAPELGGVGCGVATAIVYWIMLLLLLFYIVTSKRLAHVKVFETFHKPQPKELIRLFRLGFPVAAALFFEVTLFAVVALLVAPLGSTVVAA 276
hMATE1          ANYLFLH----QLHLGVIGSALANLISQYTLALLLFLYILGKKL--HQATWGGWSLECLQDWASFLRLAIPSMLMLCMEWWAYEVGSFLSGILGMVELGA 294
AhMATE11        LCWALVL----KYDLGSRGAAISNCISYWVNVLILALYIKFSPK--CSQTWTGFSVEAFHDIPSFLKLAIPSAVMVCLELWSFELMVLLSGRLPNPNV-- 287
                              **  *      *      * **                            *  *       *         *   *

                 TM8                              TM9                                   TM10
NorM-VC         HQVALNFSSLVFMFPMSIGAAVSIRVGHKLGEQDTKGAAIAANVGLMTGLATACITALLTVLFREQIALLYTENQVVVALAMQLLLFAAIYQCMDAVQVV 376
hMATE1          QSIVYELAIIVYMVPAGFSVAASVRVGNALGAGDMEQARKSSTVSLLITVLFAVAFSVLLLSCKDHVGYIFTTDRDIINLVAQVVPIYAVSHLFEALACT 394
AhMATE11        -----NTASTIWMIPFGLSGAISTRVSNELGAGNPWSARLAVRVVVVAAIIEGTLVAALMIMIRNVWGHVYSNNIQVVRQVGFMLPILAASNFLDGLQGV 382
                            * *     * * **   **      *     *              *                             *

                         TM11                          TM12
NorM-VC         AAGSLR--GYKDMTAIFHRTFISYWVLGLPTGYILGMTNWLTEQPLGAKGFWLGFIIGLSAAA---LMLGQRLYWLQ-----KQSDDVQLHLAAK----- 461
hMATE1          SGGVLRGSGNQKVGAIVN--TIGYYVVGLPIGIALMFA-----TTLGVMGLWSGIIICTVFQAVCFLGFIIQLNWKKACQQAQVHANLKVNNVPRSGNSA 487
AhMATE11        VSGIVRGCGWQKMGAFIN--LGSYYVVGVPSAIVLAFV-----LHLEVKGLWLGIICALVVQVLGLMIITVRIDWKK-------EAMVTMERVNNSGQAS 468
                  *  *  *     *        * * * *    *          *   * * * *                  *

NorM-VC         -----------------------------------------------------------------------------------                 461
hMATE1          LPQDPLHPGCPENLEGILTNDVGKTGEPQSDQQMRQEEPLPEHPQDGAKLSRKQLVLRRGLLLLGVFLILLVGILVRFYVRIQ                 570
AhMATE11        IER-----------------------------------------------------------VNNSFI---------------                 477
```

Figure S2. Multiple Sequence alignment of AhMATE11 with NorM-VC and hMATE1 was performed using ClustalW v2.0 with default settings. The twelve transmembrane domains (TM1–TM12) are annotated above the alignment. The conserved amino acid residues E268 (TM7) and D377 (TM10) in AhMATE11 are highlighted in red and blue, respectively.
